# Supplementary material for: An examination of force maps targeted at orientation interactions in moving groups
Source: PLoS One. 2023 Sep 7;18(9):e0286810. doi: 10.1371/journal.pone.0286810 (PMC10484433; doi:10.1371/journal.pone.0286810)
Supplement: S1 File — Supplementary Information including details on force mapping, simulation models, supplementary tables, and supplementary. Figs and results referenced in the main text. (PDF) [file pone.0286810.s002.pdf]

An examination of force maps targeted at orientation interactions in moving groups – Supplementary Information

R. K. Mudaliar[1,2,*], T. M. Schaerf[2]

1. School of Mathematical and Computing Science, Fiji National University, Fiji

2. School of Science and Technology, University of New England, Armidale, NSW 2351, Australia

* corresponding author. Email: Rajnesh.Mudaliar@fnu.ac.fj

S1 Force mapping

The baseline method for force mapping that we used and built upon for this study is derived from the approach applied by [4, 7]. The overarching goal of the following sequence of calculations is to separately map out the mean changes in speed and direction of motion of individuals as a function of the relative (x, y) coordinates of their neighbours, in a consistent frame of reference. The consistent frame of reference places a "focal" individual at the origin of the coordinate system, with their velocity vector aligned with the positive x-axis. The analysis is applied to a set of individual trajectories $(x_i(t), y_i(t))$ (for individual i at time t), discretely sampled at equally spaced time intervals of duration Δt. Details in sections S1.1 and S1.2 are largely reproduced from sections 2.1 and 2.2 of [6], and are provided for necessary context and completeness.

S1.1 Fundamental measures of movement

Initially, the components of the velocities of each individual are estimated using the standard forward-difference approximations:

$$u_i(t) = \frac{x_i(t+\Delta t) - x_i(t)}{\Delta t} \qquad \text{and} \qquad v_i(t) = \frac{y_i(t+\Delta t) - y_i(t)}{\Delta t} \tag{S1.1}$$

The components of the unit vector in the direction of an individual's velocity, $\mathbf{V}_i(t) = u_i(t)\mathbf{i} + v_i(t)\mathbf{j}$, for each individual i, for each time t, are defined as

$$\hat{u}_i(t) = \frac{u_i(t)}{|\mathbf{V}_i(t)|} \qquad \text{and} \qquad \hat{v}_i(t) = \frac{v_i(t)}{|\mathbf{V}_i(t)|} \tag{S1.2}$$

where the norm $|\mathbf{V}_i(t)|$ is

$$|\mathbf{V}_i(t)| = \sqrt{u_i(t)^2 + v_i(t)^2}. \tag{S1.3}$$

The components of velocity are used to find the magnitude of the change in direction of motion of each individual i from time t to time $t + \Delta t$ via:

$$\psi_i(t) = \cos^{-1}\left(\hat{u}_i(t)\hat{u}_i(t+\Delta t) + \hat{v}_i(t)\hat{v}_i(t+\Delta t)\right). \tag{S1.4}$$

The sense of rotation of the individual is also determined explicitly, that is, whether the individual turned clockwise or anticlockwise at each time step. To do this the sign of the vertical component of the cross product of the unit velocity vectors of each individual i at times t and $t + \Delta t$ is examined. Individual i turned anticlockwise (clockwise) as it moved from time t to time $t + \Delta t$ if the sign of the following equation is positive (negative):

$$\lambda_i(t) = \mathrm{sgn}\left(\hat{u}_i(t)\hat{v}_i(t+\Delta t) - \hat{u}_i(t+\Delta t)\hat{v}_i(t)\right). \tag{S1.5}$$

where sgn is the sign function.

Taking into account whether the individual i turns clockwise or anticlockwise, the signed change in direction of motion over time in radians is given by:

$$\frac{\Delta\theta_i}{\Delta t}(t) = \begin{cases} \lambda_i(t)\frac{\psi_i(t)}{\Delta t} & \text{if } \lambda_i(t) \neq 0, \\ \frac{\psi_i(t)}{\Delta t} & \text{if } \lambda_i(t) = 0. \end{cases} \tag{S1.6}$$

The speed of individual i at time t is estimated directly from the components of velocity via:

$$s_i(t) = \sqrt{u_i(t)^2 + v_i(t)^2}. \tag{S1.7}$$

The change in speed over time of individual i is then approximated using

$$\frac{\Delta s_i}{\Delta t}(t) = \frac{s_i(t+\Delta t) - s_i(t)}{\Delta t}. \tag{S1.8}$$

S1.2 Relative coordinates of group mates

The distance between a focal individual i (every individual is treated as a focal individual in turn as data is aggregated in a force mapping calculation) and every other individual j in the group, for all times t, is determined using the distance formula:

$$d_{i,j}(t) = \sqrt{(x_j(t) - x_i(t))^2 + (y_j(t) - y_i(t))^2}. \tag{S1.9}$$

Next, the angle between the unit velocity vector of the focal individual i and the straight line segment from individual i to individual j is calculated. The unit vector pointing along the line segment from individual i to individual j has components:

$$\hat{x}_{i,j}(t) = \frac{x_j(t) - x_i(t)}{d_{i,j}(t)} \qquad \text{and} \qquad \hat{y}_{i,j}(t) = \frac{y_j(t) - y_i(t)}{d_{i,j}(t)}. \tag{S1.10}$$

The unsigned angle between the direction of motion of individual i and the unit vector pointing from individual i to individual j is:

$$\phi_{i,j}(t) = \cos^{-1}\left(\hat{u}_i(t)\hat{x}_{i,j}(t) + \hat{v}_i(t)\hat{y}_{i,j}(t)\right). \tag{S1.11}$$

A similar technique to that summarised by equation (S1.5) is then employed to determine if individual j lies to the left or right of individual i. Relative to the direction of motion of individual i, individual j lies to the left (right) of individual i if the sign of the following equation is positive (negative):

$$\zeta_{i,j}(t) = \operatorname{sgn}\left(\hat{u}_i(t)\hat{y}_{i,j}(t) - \hat{v}_i(t)\hat{x}_{i,j}(t)\right). \tag{S1.12}$$

Taking into account whether individual j is on the left or right of individual i and combining equations (S1.11) and (S1.12), the signed angle between the direction of motion of individual i and the unit position vector of individual j relative to focal individual i is then:

$$\vartheta_{i,j}(t) = \begin{cases} \zeta_{i,j}(t)\phi_{i,j}(t) & \text{if } \zeta_{i,j}(t) \neq 0, \\ \phi_{i,j}(t) & \text{if } \zeta_{i,j}(t) = 0. \end{cases} \tag{S1.13}$$

Hence, equations (S1.9) and (S1.13) give the polar coordinates of the position of an individual j relative to individual i's position and direction of motion, $(d_{i,j}(t), \vartheta_{i,j}(t))$. These polar coordinates are converted to rectangular coordinates using:

$$\begin{aligned} x_{i,j,relative}(t) &= d_{i,j}(t)\cos\left(\vartheta_{i,j}(t)\right), \\ y_{i,j,relative}(t) &= d_{i,j}(t)\sin\left(\vartheta_{i,j}(t)\right). \end{aligned} \tag{S1.14}$$

Figures S1 and S2 illustrate some of the key measures of locomotion and relative neighbour positions associated with the construction of standard force maps.

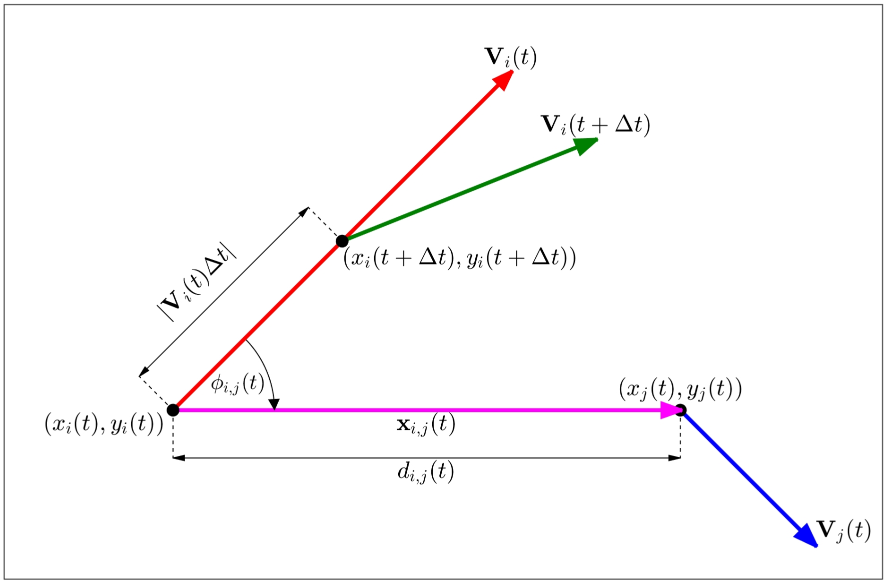

Fig S1. Variables used to describe the movement of individual i and relative coordinates of its groupmate j. The position and velocity of individual i are denoted by $(x_i(t), y_i(t))$ (black dot) and $\mathbf{V}_i(t)$ (red arrow) respectively. Δt is the time step increment. $(x_i(t+\Delta t), y_i(t+\Delta t))$ and $\mathbf{V}_i(t+\Delta t)$ (green arrow) represent the position and velocity of individual i after it has travelled from time t to time $t+\Delta t$. $|\mathbf{V}_i(t)\Delta t|$ is the distance individual i travels from time t to time $t+\Delta t$. The position and velocity of individual j are denoted by $(x_j(t), y_j(t))$ and $\mathbf{V}_j(t)$ (blue arrow) respectively. The linear distance between individual i and its groupmate j is denoted by $d_{i,j}(t)$. The vector pointing along the line segment from individual i to individual j is denoted by $\mathbf{x}_{i,j}(t)$ (magenta arrow). The unsigned angle between the direction of motion of individual i and the unit vector pointing from individual i to individual j is denoted by $\phi_{i,j}(t)$ (adapted from [8]).

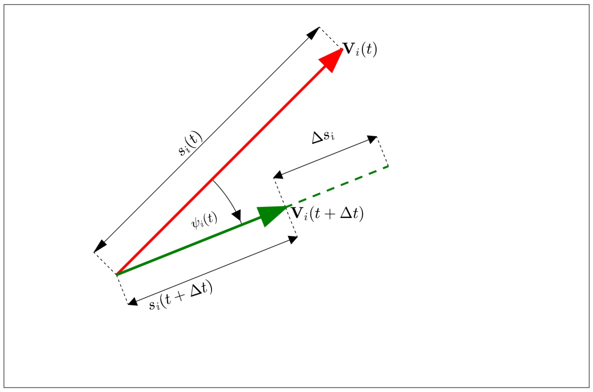

Fig S2. Variables used to describe the change in direction and speed of individual i. The speeds of individual i at time t and $t+\Delta t$ are denoted by $s_i(t)$ and $s_i(t+\Delta t)$ respectively. $\Delta s_i = s_i(t+\Delta t) - s_i(t)$ is the difference in the speed of individual i as it travels from time t to time $t+\Delta t$. $\psi_i(t)$ is the measure of the magnitude of the change in direction of motion of individual i as it travels from time t to time $t+\Delta t$, which is formed when the vectors $\mathbf{V}_i(t)$ and $\mathbf{V}_i(t+\Delta t)$ are placed tail-to-tail (adapted from [8]).

S1.3 Dimensions and use of bins in standard force mapping

In the $(x_{i,j,relative}(t), y_{i,j,relative}(t))$ relative coordinate system, the focal individual i is located at the origin, $(0,0)$, with its velocity vector aligned with the positive x-axis. A square local domain centred on the focal individual is divided into a set of overlapping square bin regions. For each focal individual i, partner j, and discrete time t, the changes in speed, $\frac{\Delta s_i}{\Delta t}(t)$, and direction of motion, $\frac{\Delta \theta_i}{\Delta t}(t)$, are then stored in all bins that contain $(x_{i,j,relative}(t), y_{i,j,relative}(t))$. Once all the data for these measures of interest are binned, the mean value of each measure in each bin is determined. More explicitly: the domain is partitioned, where $-L < x_{i,j,relative}(t) \le L, -L < y_{i,j,relative}(t) \le L$, centred on each focal individual i, into a set of overlapping square bins of side length m identified by paired column and row indices (a,b), such that the left edges and bottom edges of consecutive bins are separated by $n < m$. In other words the left edges of the bins are located at $x_{a,left} = -L, -L+n, -L+2n, ..., L-m$, the right edges of the bins are located at $x_{a,right} = -L+m, -L+m+n, -L+m+2n, ..., L$, the bottom edges of the bins are located at $y_{b,bottom} = -L, -L+n, -L+2n, ..., L-m$ and the top edges of the bins are located at $y_{b,top} = -L+m, -L+m+n, -L+m+2n, ..., L$. An example of the construction of the overlapping bins is provided in Figure S3. For all individuals i, for every time step t, and looping over all other individuals $j \ne i$ across all data from the same set and time interval, the change in direction of motion over time of focal individual i given by equation (S1.6) and the change in speed over time of focal individual i given by equation (S1.8) are placed into the bin with column and row indices (a,b), if $x_{a,left} < x_{i,j,relative}(t) \le x_{a,right}$ and $y_{b,bottom} < y_{i,j,relative}(t) \le y_{b,top}$. Once this process is complete, mean values in each

bin are determined and the resulting fitted functions are rendered via a surface plotting function (throughout this study we use MATLAB's *surf* function).

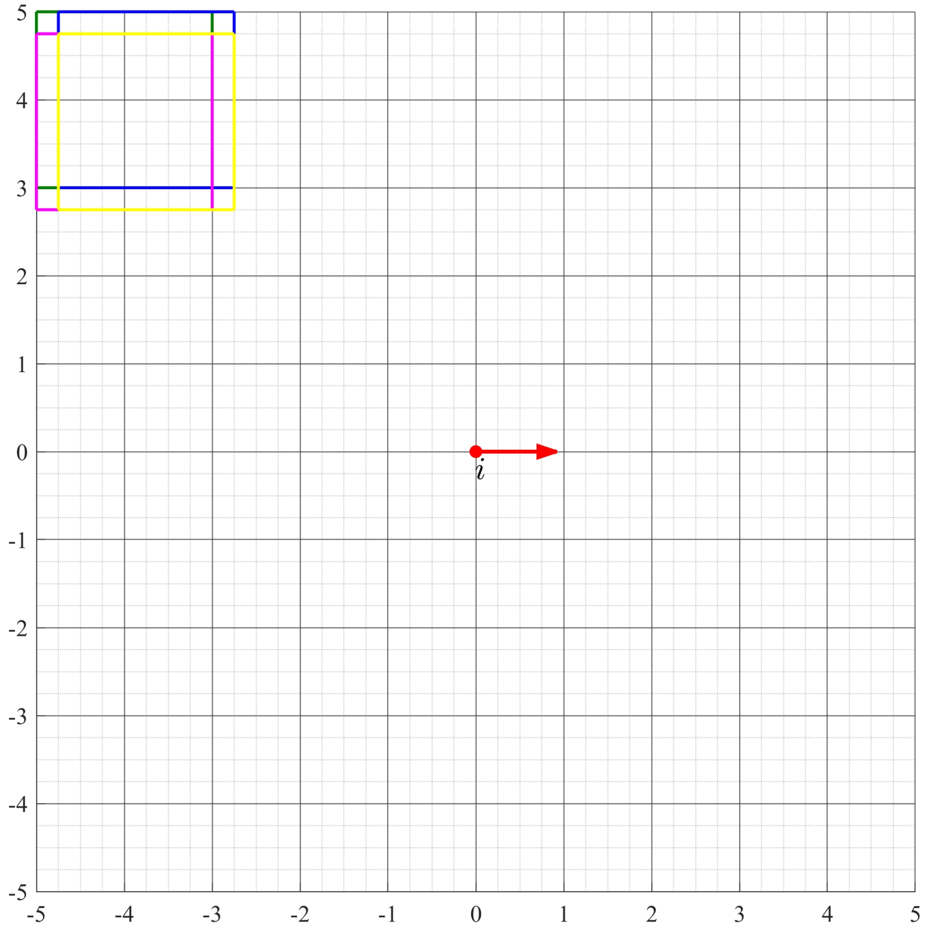

Fig S3. An example of a square local domain centered on the focal individual i divided into a set of overlapping square bin regions (including the green, yellow, blue and magenta squares). Here, $x_{a,left} = -5, -4.75, -4.5, ..., 3$, $x_{a,right} = -3, -2.75, -2.5, ..., 5$, $y_{b,bottom} = -5, -4.75, -4.5, ..., 3$ and $y_{b,top} = -3, -2.75, -2.5, ..., 5$.

S1.4 Division of local domain into bin regions for this study

We focussed on two forms of force map for this study, those that treated the relative (x, y) coordinates of neighbours and angular differences in direction of motion, $\varphi_{i,j}$ (as described in the main text), as independent variables, and those that just used neighbour distances $d_{i,j}$ and angular differences $\varphi_{i,j}$ to separate data. In both cases, the dependent

variable of interest was the change in direction of the focal individual, $\Delta\theta_i/\Delta t$. Force maps generated as a function of $(x, y, \varphi_{i,j})$ partitioned data using overlapping bins for x- and y-coordinates (as described above, with full details of bin dimensions given in [6]), but non-overlapping bins for values of $\varphi_{i,j}$ (detailed below). Force maps generated as a function of $(d_{i,j}, \varphi_{i,j})$ used overlapping bins for both distances and angular differences (detailed below). In addition, binning for the closely related local alignment plots was based on relative (x, y) coordinates of neighbours, as per [6].

a. **Non-overlapping bins for Angular difference**
We created 12 non-overlapping bins of width $\frac{\pi}{6}$ from $-\pi$ to π (in radians). The lower and upper boundaries for the fth bin were $\varphi_{f,left} = -\pi, -\pi+\frac{\pi}{6}, -\pi+\frac{\pi}{3}, ..., \pi-\frac{\pi}{3}, \pi-\frac{\pi}{6}$ and $\varphi_{f,right} = -\pi+\frac{\pi}{6}, -\pi+\frac{\pi}{3}, ..., \pi-\frac{\pi}{6}, \pi$ respectively. (Non-overlapping bins were used when examining $\frac{\Delta\theta}{\Delta t}$ as a function of $(x, y, \varphi_{i,j})$.)

b. **Overlapping bins for angular difference**
We partitioned the angular difference, where $-\pi \leq \varphi_{i,j}(t) \leq \pi$, into a set of overlapping bins with each bin length equal to $\frac{\pi}{6}$, such that the lower and upper boundaries of the consecutive bins were separated by $\frac{\pi}{24}$. In other words the lower and upper boundaries for the gth bin were $\varphi_{i,j_{(g,left)}} = -\pi, -\pi+\frac{\pi}{24}, -\pi+\frac{\pi}{12}, ..., \pi-\frac{5\pi}{24}, \pi-\frac{\pi}{6}$ and $\varphi_{i,j_{(g,right)}} = -\pi+\frac{\pi}{6}, -\pi+\frac{5\pi}{24}, -\pi+\frac{\pi}{4}, ..., \pi+\frac{\pi}{12}, \pi-\frac{\pi}{24}, \pi$ respectively. (Overlapping bins were used when examining $\frac{\Delta\theta}{\Delta t}$ as a function of $(d_{i,j}, \varphi_{i,j})$.)

c. **Overlapping bins for distance between individuals**
To create overlapping bins for $d_{i,j}$, we partitioned the distance between individuals where $0 \leq d_{i,j}(t) \leq D$, into a set of overlapping bins of side length p, such that the lower and upper boundaries of consecutive bins were separated by $q < p$. The lower and upper boundaries for the hth bin were $r_{h,left} = 0, q, 2q, ..., D-p$ and the upper boundaries of the same bins were $r_{h,right} = p, q+p, 2q+p..., D$ respectively.

S2 Models

S2.1 Orientation-only model

We encoded the model described in [9] in MATLAB with reflective boundaries, or on an open domain with no physical boundaries. In the case of reflecting boundary conditions, simulations were carried out over a square domain with side lengths L, such that $0 \leq x \leq L$ and $0 \leq y \leq L$. Initially, N individuals were randomly distributed inside this square domain for both simulations with and without boundaries. Each individual i had its own randomly assigned initial direction of motion θ_i and moved at constant speed s. At each discrete time step, t, each individual adjusts its direction of motion for the next time step, $t + \Delta t$, to approximately match the average direction of motion of all individuals within r_o units of itself (including its own direction of motion). Writing the direction of motion of individual

i at time t as $\theta_i(t)$, the average of all directions within a distance r_o of individual i at the same time is:

$$\bar{\theta}_i(t) = \text{atan2}\left(\sum_{k\in r_o} \sin(\theta_k(t)), \sum_{k\in r_o} \cos(\theta_k(t))\right), \tag{S2.1}$$

where the $k \in r_o$ term denotes the indices of all neighbours within the threshold distance r_o (including the self index i). The individual's direction of motion at the next time step is then given by $\theta_i(t + \Delta t) = \bar{\theta}_i(t) + \Delta\theta_\eta$, where $\Delta\theta_\eta$ is a normally distributed random variate with mean zero, and standard deviation η.

The components of velocity for each individual are then determined at each time step. The component of velocity in the x-direction, u, is $u_i(t + \Delta t) = s\cos(\theta(t + \Delta t))$ and the component in the y-direction, v, is $v_i(t + \Delta t) = s\sin(\theta(t + \Delta t))$. Positions of individuals are then updated via $x_i(t + \Delta t) = x_i(t) + u_i(t)\Delta t$ and $y_i(t + \Delta t) = y_i(t) + v_i(t)\Delta t$ respectively.

The choice to perform simulations with this model in both unbounded and bounded domains was made to try to generate data sets that might replicate experimental observations of fish in the wild (with an effectively unbounded domain compared to the limited field of view of a camera; see for example [10]), or aquaria-based observations with clearly defined boundaries (one of the most commonly used systems in controlled experimental studies of collective movement; see for example [4]). For the bounded domain, we implemented reflecting boundary conditions as follows. If an individual was within a threshold distance ϵ of either the left or right boundary (as illustrated in Figure (S4)), then we reversed the component of its velocity in the x-direction by multiplying u_i by -1. Similarly, if the individual was within ϵ of the upper or lower boundaries, then we reversed its component of velocity in the y-direction by multiplying v_i by -1.

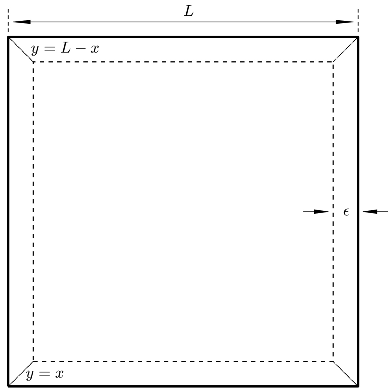

Fig S4. Bounded domain. If an individual moves within ϵ units of the left or right boundaries, then it will reverse the x-component of its velocity (u) to avoid collision with the wall. Similarly, if an individual moves within ϵ units of the top or bottom boundaries, then it will reverse the y-component of its velocity (v).

In prototype calculations with the bounded domain, we observed that individuals could escape the domain via the corners, possibly due to interactions with neighbours that

continually forced them back to, and eventually out of the corner regions. To prevent such individuals escaping from the corners we decided, if in any case individuals fall on the lines $y = L - x$ or $y = x$ when they approach the corners of the square domain, then those individuals would randomly reverse either the x- or y-component of their velocity only (with an equal probability of switching either component).

S2.1.1 Exact pairwise orientation interactions in orientation-only model

In the absence of noise, it is possible to write an exact form for the prescribed change in direction of a focal individual i in response to a single neighbour j as a function of $d_{i,j}$ and $\varphi_{i,j}$ for this model, a form convenient for comparison with the force maps studied here. Consider a focal individual i positioned at $(0, 0)$ and moving in the positive $x-$direction, parallel to the $x-$axis. The direction of motion of the focal individual i is $\mathbf{V}_i(t) = \mathbf{i}$ and the neighbour j has direction of motion $\mathbf{V}_j(t) = \cos(\theta_j(t))\mathbf{i} + \sin(\theta_j(t))\mathbf{j}$. The partner j is within the interaction/orientation distance, r_o. The sum of the directions $\mathbf{V}_i(t)$ and $\mathbf{V}_j(t)$ is:

$$\mathbf{V}_i(t) + \mathbf{V}_j(t) = (\cos(\theta_j(t)) + 1)\,\mathbf{i} + \sin(\theta_j(t))\mathbf{j}. \tag{S2.2}$$

In this alignment-only model the focal individual adjusts its motion to be the average of the directions of all individuals within the interaction distance r_o, so for a single partner the new direction of motion can be described by the vector $\mathbf{V}_i(t) + \mathbf{V}_j(t)$. To determine the signed change in angle over time for a focal individual i, we construct the unit vector in the direction of the above resultant, that is:

$$\frac{\mathbf{V}_i + \mathbf{V}_j}{|\mathbf{V}_i + \mathbf{V}_j|} = \frac{\cos(\theta_j) + 1}{\sqrt{(\cos(\theta_j) + 1)^2 + (\sin(\theta_j))^2}}\mathbf{i} + \frac{\sin(\theta_j)}{\sqrt{(\cos(\theta_j) + 1)^2 + (\sin(\theta_j))^2}}\mathbf{j}. \tag{S2.3}$$

The denominator on the right hand side of equation (S2.3) can be simplified as follows:

$$\begin{aligned}\sqrt{(\cos(\theta_j) + 1)^2 + (\sin(\theta_j))^2} &= \sqrt{\cos^2(\theta_j) + 2\cos(\theta_j) + 1 + \sin^2(\theta_j)} \\ &= \sqrt{2(\cos(\theta_j) + 1)}.\end{aligned} \tag{S2.4}$$

Hence, the unit vector in the direction of the resultant can be written as:

$$\hat{\mathbf{b}} = \frac{\mathbf{V}_i + \mathbf{V}_j}{|\mathbf{V}_i + \mathbf{V}_j|} = \frac{\cos(\theta_j) + 1}{\sqrt{2(\cos(\theta_j) + 1)}}\mathbf{i} + \frac{\sin(\theta_j)}{\sqrt{2(\cos(\theta_j) + 1)}}\mathbf{j}. \tag{S2.5}$$

The magnitude of the change in angle of motion of a focal individual i can be determined via the definition of the dot product, $\mathbf{a} \cdot \mathbf{b} = |a||b|\cos(\theta)$, as applied to the current direction of motion of the focal individual at time t ($\mathbf{i}$ in the relative coordinate system) and the new direction of motion $\hat{\mathbf{b}}$ at time $t + \Delta t$. The result is:

$$\begin{aligned}|\Delta\theta_i| &= \cos^{-1}\left(\frac{\cos(\theta_j)+1}{\sqrt{2}(\cos(\theta_j)+1)^{\frac{1}{2}}}\right) \\ &= \cos^{-1}\left(\frac{(\cos(\theta_j)+1)^{\frac{1}{2}}}{\sqrt{2}}\right) \\ &= \cos^{-1}\left(\frac{\sqrt{2}}{2}(\cos(\theta_j) + 1)^{\frac{1}{2}}\right).\end{aligned} \tag{S2.6}$$

In order to determine the sense of rotation (clockwise or anticlockwise) of the focal individual we determine

$$\begin{aligned} \mathbf{i} \times \hat{\mathbf{b}} &= \begin{vmatrix} \mathbf{i} & \mathbf{j} & \mathbf{k} \\ 1 & 0 & 0 \\ \frac{\cos(\theta_j)+1}{\sqrt{2(\cos(\theta_j)+1)}} & \frac{\sin(\theta_j)}{\sqrt{2(\cos(\theta_j)+1)}} & 0 \end{vmatrix} \\ &= 0\mathbf{i} - 0\mathbf{j} + \frac{\sin(\theta_j)}{\sqrt{2(\cos(\theta_j)+1)}}\mathbf{k}. \end{aligned} \tag{S2.7}$$

Thus, the sense of rotation of the focal individual is

$$\lambda = \operatorname{sgn}\left(\frac{\sin(\theta_j)}{\sqrt{2(\cos(\theta_j)+1)}}\right), \tag{S2.8}$$

where sgn is the sign function and λ is positive for anti-clockwise turns, negative for clockwise turns and zero if $\theta_j = \{0, \pi\}$. When $d_{i,j} < r_o$, and because i has a single partner j, it follows that $\varphi_{i,j} = \theta_j$. As noted earlier in the main paper, $\varphi_{i,j}$ (equation (2.1)) is the difference in directions of motion between individuals i and j. Taking into account whether the individual i turns clockwise or anticlockwise, the signed changes in direction of a focal individual i with a group mate j as a function of $d_{i,j}$ and $\varphi_{i,j}$ is given by:

$$\frac{\Delta\theta_i}{\Delta t} = \begin{cases} 0 & \text{if } d_{i,j} > r_o \\ \lambda\frac{|\Delta\theta_i|}{\Delta t} & \text{if } d_{i,j} \leq r_o \text{ and } \lambda \neq 0, \\ \frac{|\Delta\theta_i|}{\Delta t} & \text{if } d_{i,j} \leq r_o \text{ and } \lambda = 0. \end{cases} \tag{S2.9}$$

$\frac{\Delta\theta_i}{\Delta t}$ in equation (S2.9) is not defined for $\theta_j = \pi$ (or 180°). This is because equations (S2.3), (S2.5) and (S2.6) (and later derived expressions) are not defined when $\theta_j = 180°$. This is due to the ambiguity of determining the average of two directions that differ by 180°.

S2.1.2 Simulations and additional analysis of orientation-only model data

Simulations were performed for 1000 time steps with time step $\Delta t = 1$. Initially the individuals were randomly orientated with random directions of motion inside a square of length L=7 (contained within the square boundary for simulations in a bounded domain). Simulations were carried out for varying interaction radii r_o at intervals of 0.5 from 0.5 to 2.5 units and for varying group sizes with $N \in \{4, 6, 8, 10, 12, 14, 16, 32\}$. The number of simulations performed for each parameter set is listed in Table S2. As listed in Table S2, we needed to perform more than 10 simulations for some pairs of N and r_o to provide sufficient data for subsequent calculations to approximate the area of regions of high alignment (and low variation in alignment/high focus about the mean alignment) in local alignment plots, as detailed below. Simulated group sizes were chosen to be similar to, or the same order of magnitude, as those used in trajectory based empirical studies of fish (see for example

[2, 7, 10]). Standard model output was time series of (x, y) coordinates (position data). The position data for each set of simulations was saved and then analysed via force mapping and local alignment graphs.

Table S1. Summary of parameters used in alignment only model.

Model Parameters			
Parameter	Unit	Symbol	Values Used
Number of individuals	None	N	4, 6, 8, 10, 12, 14, 16, 32
Interaction radius	units	r_o	0.5, 1, 1.5, 2, 2.5
Individual speed	Units per second	s	0.03
Time step increment	Seconds	Δt	1
The standard deviation in noise	rads	η	0.1
The length of square domain	units	L	7

Table S2. Summary of all simulations used to generate data using alignment only model.

N	r_o	number of simulations	
		unbounded domain	bounded domain
4	0.5	50	10
	1	30	10
	1.5	50	10
	2	50	30
	2.5	20	30
6	0.5	50	10
	1	10	10
	1.5	10	10
	2	10	10
	2.5	10	10
8	0.5	10	10
	1	10	10
	1.5	10	10
	2	20	20
	2.5	10	20
10	0.5	10	10
	1	10	10
	1.5	20	10
	2	10	10
	2.5	10	20
12	0.5	10	10
	1	10	10
	1.5	10	10
	2	10	10
	2.5	10	30
14	0.5	10	10
	1	10	10
	1.5	10	10
	2	10	20
	2.5	10	20
16	0.5	10	10
	1	10	10
	1.5	10	10
	2	10	10
	2.5	10	10
32	0.5	10	10
	1	10	10
	1.5	10	10
	2	10	10
	2.5	20	20

S2.2 Zonal model

Here we provide an overview of our implementation of the zonal model where individuals travel at constant speed in two dimensions, derived from Couzin et al. [1] and largely reproduced from our description in section S2 of [6]. In the model, the region surrounding each individual is divided into three non-overlapping zones bounded by concentric circles. These zones, ordered from the innermost region to the outermost, are the zone of repulsion (ZOR), the zone of orientation (ZOO), and the zone of attraction (ZOA).

S2.2.1 Initial conditions

In general, N individuals were initially distributed randomly inside the arbitrary square with $-\sqrt{2}r_a \leq x \leq \sqrt{2}r_a$ and $-\sqrt{2}r_a \leq y \leq \sqrt{2}r_a$, where r_a was the radius of the outermost boundary of the zone of attraction. The position of each individual i is denoted via its position vector $\mathbf{c}_i(t) = x_i(t)\mathbf{i} + y_i(t)\mathbf{j}$. The velocity of an individual at each time t is defined as $\mathbf{V}_i(t) = u_i(t)\mathbf{i} + v_i(t)\mathbf{j}$ and the individual's direction (unit velocity) vector is defined as $\hat{\mathbf{V}}_i(t) = \hat{u}_i(t)\mathbf{i} + \hat{v}_i(t)\mathbf{j}$, where $\hat{u}_i(t) = u_i(t)/\sqrt{u_i^2(t) + v_i^2(t)}$ and $\hat{v}_i(t) = v_i(t)/\sqrt{u_i^2(t) + v_i^2(t)}$. The initial velocity of the individuals was assigned as $\mathbf{V}_i(0) = u_i(0)\mathbf{i} + v_i(0)\mathbf{j}$, where $u_i(0) = s\cos(\theta)$, $v_i(0) = s\sin(\theta)$, θ was a uniformly distributed random variable on $(0, 2\pi)$, and s was the constant speed of all individuals.

S2.2.2 Rules of interaction

When neighbours are in a focal individual i's ZOR, then the focal individual will act preferentially to avoid collisions by turning away from these neighbours. To enact turning based avoidance, each individual i that has group mates in its ZOR at time t prefers that its own direction of motion at time $t + \Delta t$ is the negative of the average direction to neighbouring individuals in the ZOR at time t, that is

$$\mathbf{d}_{r_i}(t + \Delta t) = -\sum_{j \in \text{ZOR},\, j \neq i} \frac{\mathbf{r}_{i,j}(t)}{|\mathbf{r}_{i,j}(t)|}. \tag{S2.10}$$

where $\mathbf{r}_{i,j}(t) = x_{i,j}(t)\mathbf{i} + y_{i,j}(t)\mathbf{j}$ is the vector from the position of individual i to the position of individual j. The total number of individuals in individual i's ZOR at time t is denoted as n_r. The unit direction vector associated with an individual's preferred direction of motion based on repulsion interactions is $\hat{\mathbf{d}}_{r_i}(t + \Delta t) = \frac{\mathbf{d}_{r_i}(t+\Delta t)}{|\mathbf{d}_{r_i}(t+\Delta t)|}$.

If there are no neighbours in individual i's zone of repulsion then that individual will respond to the individuals present in its ZOO and ZOA. The number of detectable neighbours in individual i's ZOO is denoted by n_o; such neighbours satisfy the condition that $r_r \leq |\mathbf{r}_{i,j}| < r_o$. Similarly, the number of detectable neighbours in individual i's ZOA is denoted by n_a; these neighbours satisfy the condition that $r_o \leq |\mathbf{r}_{i,j}| < r_a$. The radial width of the ZOO is $\Delta r_o = r_o - r_r$ and the radial width of the ZOA is $\Delta r_a = r_a - r_o$.

Individuals prefer to align their direction of motion with that of neighbours in their ZOO. This preferred direction based on orientation interactions is given by

$$\mathbf{d}_{o_i}(t+\Delta t) = \sum_{j\in \text{ZOO}} \frac{\mathbf{V}_j(t)}{|\mathbf{V}_j(t)|}. \tag{S2.11}$$

where $|\mathbf{V}_j(t)| = \sqrt{(u_j(t))^2 + (v_j(t))^2}$. The associated unit direction vector is $\hat{\mathbf{d}}_{o_i}(t+\Delta t) = \frac{\mathbf{d}_{o_i}(t+\Delta t)}{|\mathbf{d}_{o_i}(t+\Delta t)|}$.

Individuals will attempt to move towards neighbours in their ZOA, with their preferred direction of motion based on attraction interactions alone given by

$$\mathbf{d}_{a_i}(t+\Delta t) = \sum_{j\in \text{ZOA}} \frac{\mathbf{r}_{i,j}(t)}{|\mathbf{r}_{i,j}(t)|}. \tag{S2.12}$$

The associated unit direction vector for movement towards neighbours in an individual's ZOA is $\hat{\mathbf{d}}_{a_i}(t+\Delta t) = \frac{\mathbf{d}_{a_i}(t+\Delta t)}{|\mathbf{d}_{a_i}(t+\Delta t)|}$.

The following five cases are considered to determine the final intended direction $\mathbf{d}_i(t+\Delta t)$ for each individual i:

- Case 1: if $n_r \neq 0$ then $\mathbf{d}_i(t+\Delta t) = \hat{\mathbf{d}}_{r_i}(t+\Delta t)$. If not, go to Case 2.
- Case 2: if $n_o \neq 0$ and $n_r = 0$ and $n_a = 0$ then $\mathbf{d}_i(t+\Delta t) = \hat{\mathbf{d}}_{o_i}(t+\Delta t)$. If not, go to Case 3.
- Case 3: if $n_a \neq 0$ and $n_r = 0$ and $n_o = 0$ then $\mathbf{d}_i(t+\Delta t) = \hat{\mathbf{d}}_{a_i}(t+\Delta t)$. If not, go to Case 4.
- Case 4: if $n_o \neq 0$ and $n_a \neq 0$ and $n_r = 0$ then $\mathbf{d}_i(t+\Delta t) = \frac{1}{2}(\hat{\mathbf{d}}_{o_i}(t+\Delta t) + \hat{\mathbf{d}}_{a_i}(t+\Delta t))$. If not, go to Case 5.
- Case 5: if $n_o = 0$ and $n_a = 0$ and $n_r = 0$ then $\mathbf{d}_i(t+\Delta t) = \hat{\mathbf{V}}_i(t)$.

Then the unit final intended direction vector is calculated as $\hat{\mathbf{d}}_i(t+\Delta t) = \frac{\mathbf{d}_i(t+\Delta t)}{|\mathbf{d}_i(t+\Delta t)|} = \hat{d}_{x_i}(t+\Delta t)\mathbf{i} + \hat{d}_{y_i}(t+\Delta t)\mathbf{j}$. A small amount of noise $\Delta\theta_\eta$ is added to each component of the unit final intended direction vector with the resulting noise affected direction given by $\mathbf{d}'_i(t+\Delta t) = (\hat{d}_{x_i}(t+\Delta t) + \Delta\theta_\eta)\mathbf{i} + (\hat{d}_{y_i}(t+\Delta t) + \Delta\theta_\eta)\mathbf{j}$. $\Delta\theta_\eta$ is a normally distributed random variate with mean equal to zero, and a standard deviation of η, where η is usually a relatively small positive number. Once the stochastic element is applied to an individual's preferred direction of motion, $\mathbf{d}'_i(t+\Delta t)$ is normalized to obtain $\hat{\mathbf{d}}'_i(t+\Delta t)$.

Next, the turning rate of each individual is limited by setting a maximum turning rate of $\theta_{\Delta t}$ per time step. This is done by finding the angle $\beta_i(t+\Delta t)$ between $\hat{\mathbf{V}}_i(t)$ and $\hat{\mathbf{d}}'_i(t+\Delta t)$, that is

$$\beta_i(t+\Delta t) = \arccos(\hat{\mathbf{V}}_i(t) \cdot \hat{\mathbf{d}}'_i(t+\Delta t)). \tag{S2.13}$$

If $\beta_i(t+\Delta t) \leq \theta_{\Delta t}$ then $\hat{\mathbf{V}}_i(t+\Delta t)$ is set to $\hat{\mathbf{V}}_i(t+\Delta t) = \hat{\mathbf{d}}'_i(t+\Delta t)$. If not, then $\hat{\mathbf{V}}_i(t)$ is rotated by an angle of $\theta_{\Delta t}$ towards $\hat{\mathbf{d}}'_i(t+\Delta t)$. To do this, the following steps are applied:

i Calculate $\mathbf{a}_i(t+\Delta t)$, the component of $\hat{\mathbf{d}}'_i(t+\Delta t)$ perpendicular to $\hat{\mathbf{V}}_i(t)$ using

$$\mathbf{a}_i(t+\Delta t) = \hat{\mathbf{d}}'_i(t+\Delta t) - \left(\left(\hat{\mathbf{V}}_i(t)\right)\left(\hat{\mathbf{V}}_i(t)\cdot\hat{\mathbf{d}}'_i(t+\Delta t)\right)\right). \tag{S2.14}$$

ii Determine the unit vector $\hat{\mathbf{a}}_i(t+\Delta t) = \frac{\mathbf{a}_i(t+\Delta t)}{|\mathbf{a}_i(t+\Delta t)|}$.

iii Calculate the new direction using

$$\hat{\mathbf{V}}_i(t+\Delta t) = \cos(\theta_{\Delta t})\hat{\mathbf{V}}_i(t) + \sin(\theta_{\Delta t})\hat{\mathbf{a}}_i(t+\Delta t). \tag{S2.15}$$

Finally the position of the individual at the next time step is computed via:

$$\mathbf{c}_i(t+\Delta t) = \mathbf{c}_i(t) + s\Delta t\hat{\mathbf{V}}_i(t+\Delta t). \tag{S2.16}$$

S2.2.3 Exact pairwise orientation interactions in zonal model

In [6], exact equation forms for interactions with a single neighbour in either the zone of repulsion or zone of attraction were determined. Here we expand upon that analysis to construct an exact equation that describes a direction change based on interaction with a single neighbour in the zone of orientation.

Within the zonal model described above, consider a focal individual i positioned at $(0,0)$ and moving in the positive $x-$direction, parallel to the $x-$axis. The direction of motion of the focal individual i is $\mathbf{V}_i(t) = \mathbf{i}$ and the partner j has direction of motion $\mathbf{V}_j(t) = \cos(\theta_j(t))\mathbf{i} + \sin(\theta_j(t))\mathbf{j}$. The focal individual i prefers to align its direction of motion with that of its single partner in its zone of orientation. This preferred direction based on orientation interactions is given by

$$d_{o_i}(t+\Delta t) = \frac{\mathbf{V}_j(t)}{|\mathbf{V}_j(t)|}. \tag{S2.17}$$

The magnitude of the preferred change in angle of motion of a focal individual i is

$$|\Delta\theta_i| = \cos^{-1}\left(\cos(\theta_j)\right) = \theta_j. \tag{S2.18}$$

In order to determine the sense of rotation of the focal individual associated with the above angle, we determine

$$\begin{aligned}\mathbf{V}_i \times \mathbf{V}_j &= \begin{vmatrix} \mathbf{i} & \mathbf{j} & \mathbf{k} \\ 1 & 0 & 0 \\ \cos(\theta_j) & \sin(\theta_j) & 0 \end{vmatrix} \\ &= 0\mathbf{i} - 0\mathbf{j} + \sin(\theta_j)\mathbf{k}.\end{aligned} \tag{S2.19}$$

Thus, the sense of rotation of the focal individual is

$$\lambda = \mathrm{sgn}(\sin(\theta_j)), \tag{S2.20}$$

$d_{i,j}$ is the distance between the individual i and j and $\varphi_{i,j}$ (equation (2.1) in the main text) is the difference in directions of motion between individuals i and j. When $r_r < d_{i,j} \leq r_o$, then $\varphi_{i,j} = \theta_j$. Taking into account whether the individual i turns clockwise or anticlockwise, the signed change in direction of motion over time is given by:

$$\frac{\Delta\theta_i}{\Delta t} = \begin{cases} \lambda \frac{|\Delta\theta_i|}{\Delta t} & \text{if } r_r < d_{i,j} \leq r_o \text{ and } \lambda \neq 0, \\ \frac{|\Delta\theta_i|}{\Delta t} & \text{if } r_r < d_{i,j} \leq r_o \text{ and } \lambda = 0. \end{cases} \tag{S2.21}$$

$\frac{\Delta\theta_i}{\Delta t}$ is constrained by the maximum turning rate (in practice, 4 degress per time step (40 degrees per second) throughout our simulations).

Combining the above result with those obtained for repulsion and attraction interactions in [6], a focal individual, i, will adjust its direction of motion in response to a single neighbour, j, with relative coordinates (x_j, y_j) (in the frame of reference where the focal individual is at the origin $(0,0)$ and has direction of motion $\theta_i = 0$ (that is, aligned with the positive x-axis)) and direction of motion θ_j according to:

$$\frac{\Delta\theta_i}{\Delta t}(x_j, y_j, \theta_j) = \begin{cases} \dfrac{\operatorname{sgn}(-y_j)\cos^{-1}\left(\frac{-x_j}{\sqrt{x_j^2+y_j^2}}\right)}{\Delta t} & \text{if } x_j^2 + y_j^2 \leq r_r \text{ and } y_j \neq 0, \\ \dfrac{\cos^{-1}\left(\frac{-x_j}{\sqrt{x_j^2+y_j^2}}\right)}{\Delta t} & \text{if } x_j^2 + y_j^2 \leq r_r \text{ and } y_j = 0, \\ \dfrac{\operatorname{sgn}(\sin\theta_j)\,\theta_j}{\Delta t} & \text{if } r_r < x_j^2 + y_j^2 \leq r_o \text{ and } \operatorname{sgn}(\sin\theta_j) \neq 0, \\ \dfrac{\theta_j}{\Delta t} & \text{if } r_r < x_j^2 + y_j^2 \leq r_o \text{ and } \operatorname{sgn}(\sin\theta_j) = 0, \\ \dfrac{\operatorname{sgn}(y_j)\cos^{-1}\left(\frac{x_j}{\sqrt{x_j^2+y_j^2}}\right)}{\Delta t} & \text{if } r_o < x_j^2 + y_j^2 \leq r_a \text{ and } y_j \neq 0, \\ \dfrac{\cos^{-1}\left(\frac{x_j}{\sqrt{x_j^2+y_j^2}}\right)}{\Delta t} & \text{if } r_o < x_j^2 + y_j^2 \leq r_a \text{ and } y_j = 0. \end{cases}$$

S2.2.4 Zonal model simulations

We used existing simulation data for the zonal model, as had been generated previously for [6]. Following [6], Tables S3 and S4 list the main parameters and the values used for

our sets of simulations using the zonal model. Table S4 provides more specific details with respect to the widths of the repulsion, orientation and attraction zones for given simulation sets, along with details of the extent of the blind zone, and the emergent pattern of behaviour. Most simulations of the zonal model used in this study were run for 1000 time steps. However, longer duration simulations of 10000 time steps were also analysed in [6], and we examine analysis of these longer duration simulations in comparison to those of 1000 steps here as well. Emergent behaviour was classified as parallel motion or cohesion without parallel motion (a mixture of swarming and milling behaviour) via visual inspection, as per [6]. For the detailed analyses of zonal model simulations of groups of $N = 10$ presented in both the main paper and this supplementary information, we performed sets of 80 simulations for each set of parameter values, model details (such as presence and extent of the blind zone), broad emergent pattern of motion (parallel motion or cohesive motion), and simulation duration (1000 or 10000 time steps).

Table S3. Summary of parameters used in zonal model simulations, adapted from Table 1 in [6].

Zonal Model Parameters			
Parameter	Unit	Symbol	Values Used
Number of individuals	None	N	10, 25, 40
Zone of repulsion	units	r_r	0.5, 1, 1.5, 2
Zone of orientation	units	Δr_o	0.01, 1, 1.5, 2, 4.5, 5, 5.5
Zone of attraction	units	Δr_a	8, 11, 12.99
Blind angle	Degrees	ω_{blind}	0, 90
Maximum turning rate	Degrees per time step	$\theta_{\Delta t}$	4 (40 degrees per second)
Individual speed	Units per second	s	3
Time step increment	Seconds	Δt	0.1
The standard deviation in noise	Applied to Cartesian components of velocity/Spatial Units	η	0.1

Table S4. Summary of zone size, size of blind region and respective emergent collective behaviour for zonal model simulations run over 1000 time steps, adapted from Table 2 in [6].

r_r	Δr_o	Δr_a	Form of blind zone as given	Emergent Pattern
0.5	0.51	12.99	In ZOO, ZOA and ZOR $\omega_{blind} = 90°$	cohesion
0.5	2.5	11	In ZOO, ZOA and ZOR $\omega_{blind} = 90°$	cohesion
0.5	5.5	8	In ZOO, ZOA and ZOR $\omega_{blind} = 90°$	cohesion/ parallel aligned
1	0.01	12.99	In ZOO, ZOA and ZOR $\omega_{blind} = 90°$	cohesion
1	2	11	In ZOO, ZOA and ZOR $\omega_{blind} = 90°$	cohesion
1	5	8	In ZOO, ZOA and ZOR $\omega_{blind} = 90°$	cohesion/ parallel aligned
1.5	1.5	11	In ZOO, ZOA and ZOR $\omega_{blind} = 90°$	cohesion
1.5	4.5	8	In ZOO, ZOA and ZOR $\omega_{blind} = 90°$	cohesion/ parallel aligned
2	1	11	In ZOO, ZOA and ZOR $\omega_{blind} = 90°$	cohesion
0.5	0.51	12.99	In ZOO, ZOA and ZOR $\omega_{blind} = 0°$	cohesion
0.5	2.5	11	In ZOO, ZOA and ZOR $\omega_{blind} = 0°$	cohesion/ parallel aligned
0.5	5.5	8	In ZOO, ZOA and ZOR $\omega_{blind} = 0°$	parallel aligned
1	0.01	12.99	In ZOO, ZOA and ZOR $\omega_{blind} = 0°$	cohesion
1	2	11	In ZOO, ZOA and ZOR $\omega_{blind} = 0°$	cohesion
1	5	8	In ZOO, ZOA and ZOR $\omega_{blind} = 0°$	parallel aligned
1.5	1.5	11	In ZOO, ZOA and ZOR $\omega_{blind} = 0°$	cohesion
1.5	4.5	8	In ZOO, ZOA and ZOR $\omega_{blind} = 0°$	cohesion/ parallel aligned
2	1	11	In ZOO, ZOA and ZOR $\omega_{blind} = 0°$	cohesion
0.5	0.51	12.99	In ZOO, ZOA $\omega_{blind} = 90°$, in ZOR $\omega_{blind} = 0°$	cohesion
0.5	2.5	11	In ZOO, ZOA $\omega_{blind} = 90°$, in ZOR $\omega_{blind} = 0°$	cohesion
0.5	5.5	8	In ZOO, ZOA $\omega_{blind} = 90°$, in ZOR $\omega_{blind} = 0°$	cohesion/ parallel aligned
1	0.01	12.99	In ZOO, ZOA $\omega_{blind} = 90°$, in ZOR $\omega_{blind} = 0°$	cohesion
1	2	11	In ZOO, ZOA $\omega_{blind} = 90°$, in ZOR $\omega_{blind} = 0°$	cohesion
1	5	8	In ZOO, ZOA $\omega_{blind} = 90°$, in ZOR $\omega_{blind} = 0°$	cohesion/ parallel aligned
1.5	1.5	11	In ZOO, ZOA $\omega_{blind} = 90°$, in ZOR $\omega_{blind} = 0°$	cohesion
1.5	4.5	8	In ZOO, ZOA $\omega_{blind} = 90°$, in ZOR $\omega_{blind} = 0°$	cohesion/ parallel aligned
2	1	11	In ZOO, ZOA $\omega_{blind} = 90°$, in ZOR $\omega_{blind} = 0°$	cohesion

S2.2.5 ODE model

The final model we chose for our examintion of force mapping's efficacy was the ODE model described in [3] due to the fact that it has built-in mechanisms for increases and decreases in individual speed, and changes in direction, that reasonably resemble those that have been inferred via force mapping for real animals, specifically fish (for changes in speed, compare Figure SF2 in [6] with Figures 1C and S12 in [5], Figure 2A in [4] and Figure 2 in [7]; for changes in direction compare Figure SF3 in [6] with Figures 1D and S12 in [5] and Figure 3 in [7]). The model traces the movement of N individuals whose changes in position and velocity are described by:

$$\begin{cases} \frac{d\mathbf{x}_i}{dt} = \mathbf{v}_i, \quad (i = 1, ..., N) \\ \frac{d\mathbf{v}_i}{dt} = (\alpha - \beta|\mathbf{v}_i|^2)\mathbf{v}_i - \frac{1}{N}\sum_{j\neq i} \nabla U(|\mathbf{x}_i - \mathbf{x}_j|), \quad (i = 1, ..., N). \end{cases} \tag{S2.22}$$

where $\mathbf{x}_i$ and $\mathbf{v}_i$ are the position and velocity of the i-th individual respectively. α and β are nonnegative parameters, α models the self-propulsion of individual i and β is the friction parameter for individual i. U in the above system of ODEs is the *Morse potential* [3], defined by

$$U(|\mathbf{x}_i - \mathbf{x}_j|) = -C_A e^{\frac{-|\mathbf{x}_i - \mathbf{x}_j|}{l_A}} + C_R e^{\frac{-|\mathbf{x}_i - \mathbf{x}_j|}{l_R}}, \tag{S2.23}$$

where $|\mathbf{x}_i - \mathbf{x}_j| = \sqrt{(x_j - x_i)^2 + (y_j - y_i)^2}$. C_A and C_R are the amplitudes of the attraction and repulsion effects, and l_A and l_R relate to the ranges of attraction and repulsion respectively. The initial speed of all individuals is $\sqrt{\alpha/\beta}$.

It is possible to write down exact changes in velocity within this model in response to a single neighbour in a manner analogous to that provided for the orientation-only and zonal models earlier in this section (see section S3.2 in [6]). However, this exact form only relates to attraction- and replusion-like behaviour, as the model has no direct mechanism for orienting with neighbours.

S2.2.6 ODE model simulations

As with the zonal model, we used existing data generated via the ODE model from previous work in [6]. Table S5 lists the various sets of simulations performed for the ODE model. Simulations were performed with ten individuals ($N = 10$) for 10000 time steps with time step $\Delta t = 0.1$ for the bulk of the calculations, with time integration of the system via a standard explicit fourth order Runge-Kutta scheme (as noted in [6]). Individuals were initially uniformly randomly distributed within a square region of side length 100 units, with a uniformly distributed random direction of motion on $(0, 2\pi)$, and initial speed set to $\sqrt{\alpha/\beta}$, for all simulations. Emergent patterns of motion were identified via visual inspection of simulation animations.

Table S5. Modelling parameters and emergent collective motion patterns for the ODE model as detailed in section S2.2.5 (with $N = 10$ individuals). For item (a) a double mill is an annular structure where group members simultaneously traversed the annulus in clockwise and anticlockwise directions. For item (b), emergent states were dependent on initial conditions, and it was possible to generate an anticlockwise mill, clockwise mill and swarm. For item (b) 80 simulations were performed for each sense of milling pattern and 40 simulations were performed for swarms. For item (c), initial condition dependent emergent states were parallel aligned motion and swarm-like behaviour; we performed simulations until we had data for 80 parallel aligned groups, and 40 swarm-like groups. This data is reproduced from Table 3 in [6].

item	α	β	C_A	C_R	l_A	l_R	number of simulations	Emergent Behaviour
a	0.15	0.05	100	50	100	20	10	double mill
b	0.04	0.005	100	150	100	3	80, 80, 40	anticlockwise mill, clockwise mill, swarm
c	1	1	100	50	50	5	80, 40	parallel aligned, swarm
d	1	0.5	100	50	200	30	80	swarm

S3 Supplementary Results and Figures

S3.1 Plots from analysis of alignment only model

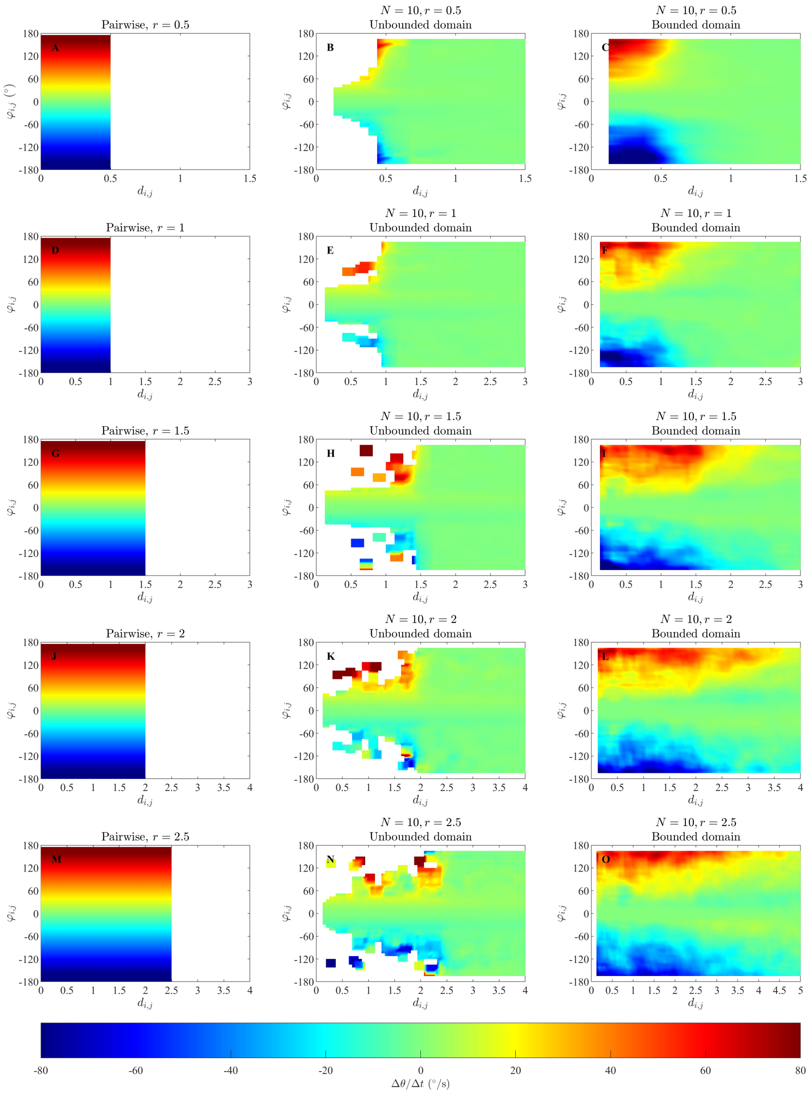

Fig S5. Left column: analytical pairwise interactions for given parameter values, as described in section S2.1.1, where turning of the individual is governed by equation (S2.9). Middle column and right column illustrates mean changes in direction over time of individuals as a function of both distance to other group mates and the differences in directions of motion between a focal individual and group mate obtained via force mapping analysis of simulations from the alignment only model with unbounded and bounded domains respectively. Positive turns are anticlockwise (redder regions) and negative turns are clockwise (bluer regions). White regions indicate that no partner individuals were recorded with the corresponding $(d_{i,j}, \varphi_{i,j})$ values. If the angular difference between focal individual and partner is positive, then the focal individual turns left to match directions with its partners (on average). If the angular difference is negative, then the focal individual turns right to match directions with is partners. $\varphi_{i,j}$ is converted to degrees.

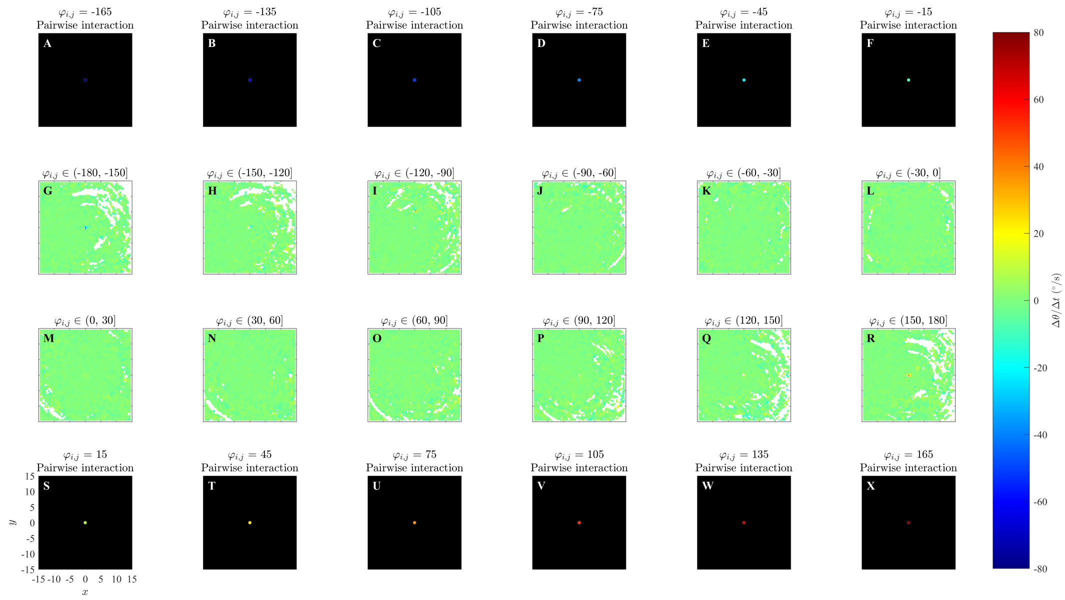

Fig S6. Analytical pairwise interactions and results of force mapping analysis of simulated data from the alignment only model with $r_o = 0.5$ and $N = 10$ in an unbounded domain. All other details are the same as for Figure 3 in the main text.

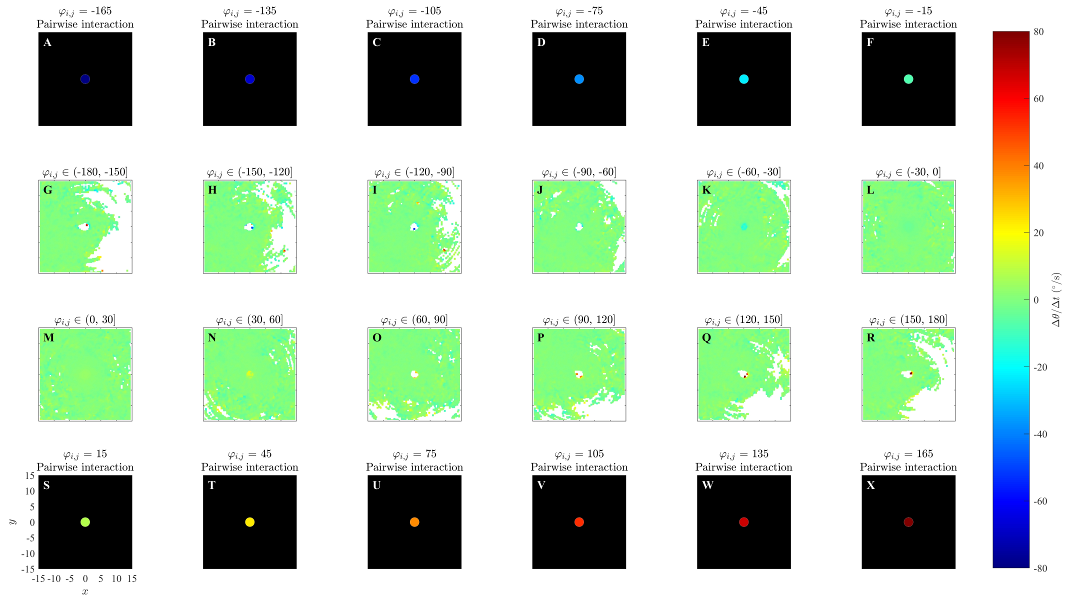

Fig S7. Analytical pairwise interactions and results of force mapping analysis of simulated data from the alignment only model with $r_o = 1.5$ and $N = 10$ in an unbounded domain. All other details are the same as for Figure 3 in the main text.

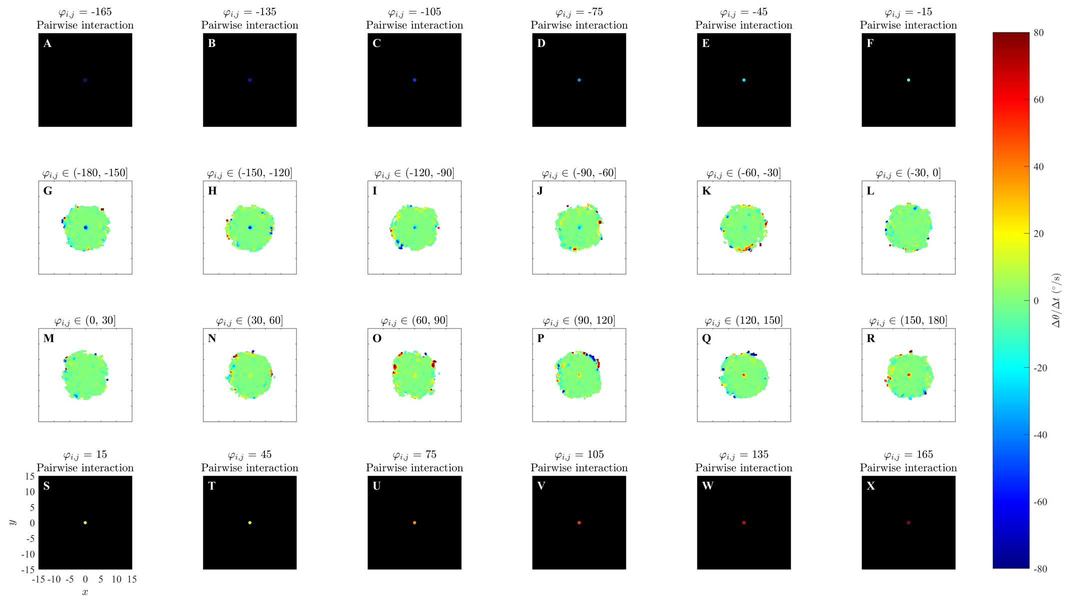

Fig S8. Analytical pairwise interactions and results of force mapping analysis of simulated data from the alignment only model with $r_o = 0.5$ and $N = 10$ in a bounded domain. All other details are the same as for Figure 3 in the main text.

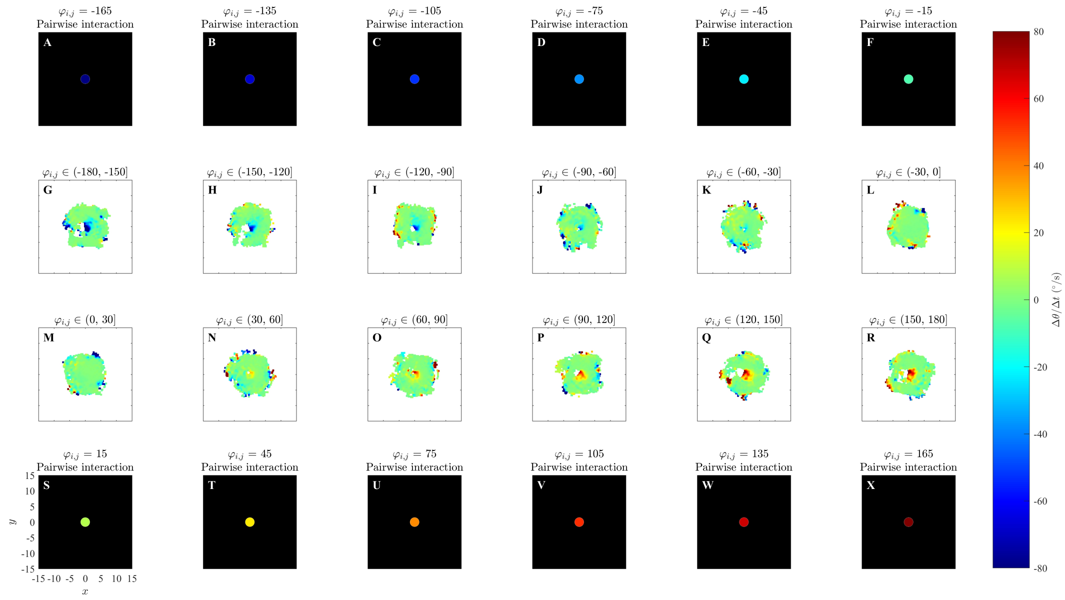

Fig S9. Analytical pairwise interactions and results of force mapping analysis of simulated data from the alignment only model with $r_o = 1.5$ and $N = 10$ in a bounded domain. All other details are the same as for Figure 3 in the main text.

S3.2 Plots from analysis of zonal model

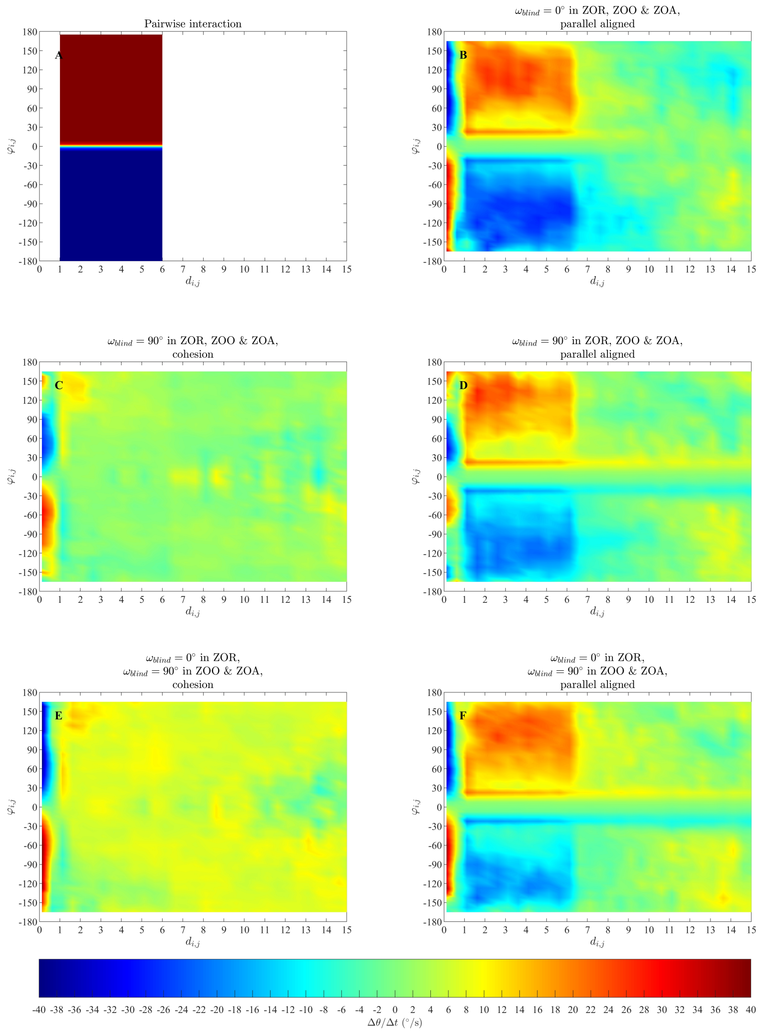

Fig S10. Panel A: analytical pairwise interactions for given parameter values, as described in section S2.2.3, where turning of the individual is governed by equation (S2.21). Panels B - F illustrates mean changes in direction over time of individuals as a function of both distance to other group mates and the differences in directions of motion between a focal individual and group mate for simulations of the zonal model with $r_r = 1$, $\Delta r_o = 5$ and $\Delta r_a = 8$. Positive values of $\frac{\Delta\theta}{\Delta t}$ correspond to anti-clockwise turns, and negative values of $\frac{\Delta\theta}{\Delta t}$ correspond to clockwise turns.

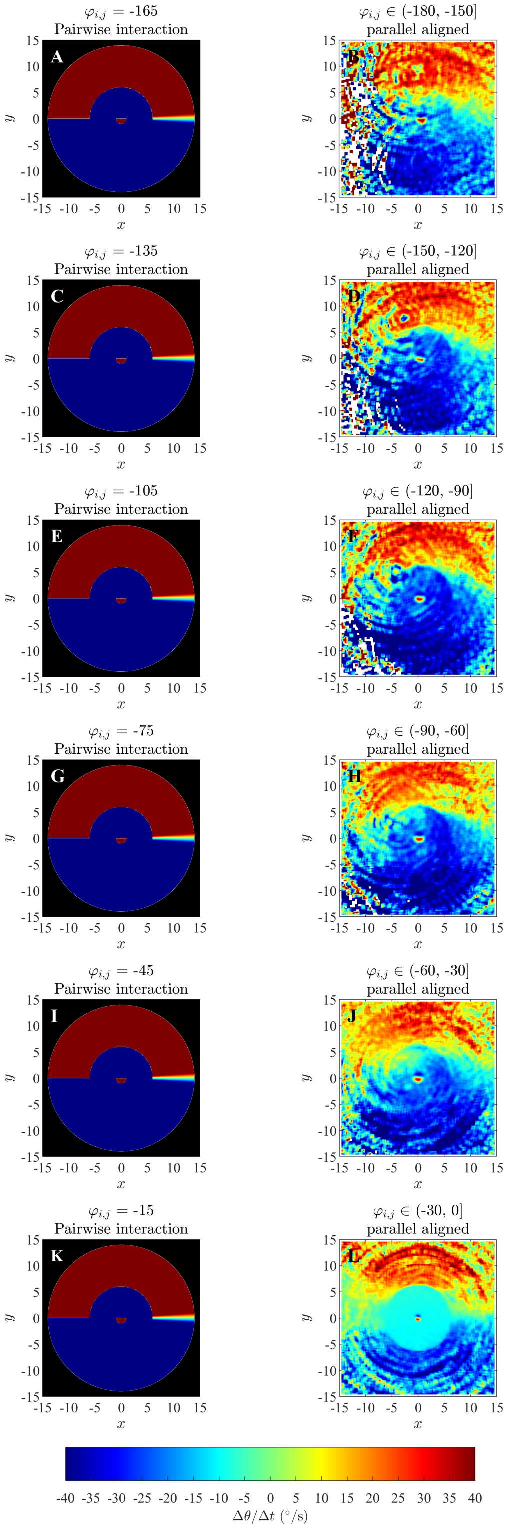

Fig S11. Left column: analytical pairwise changes in direction as prescribed by the zonal model with $r_r = 1; \Delta r_o = 5; \Delta r_a = 8; \varphi_{i,j} \leq 0$ and $\omega_{blind} = 0°$. Right column: results of force mapping.

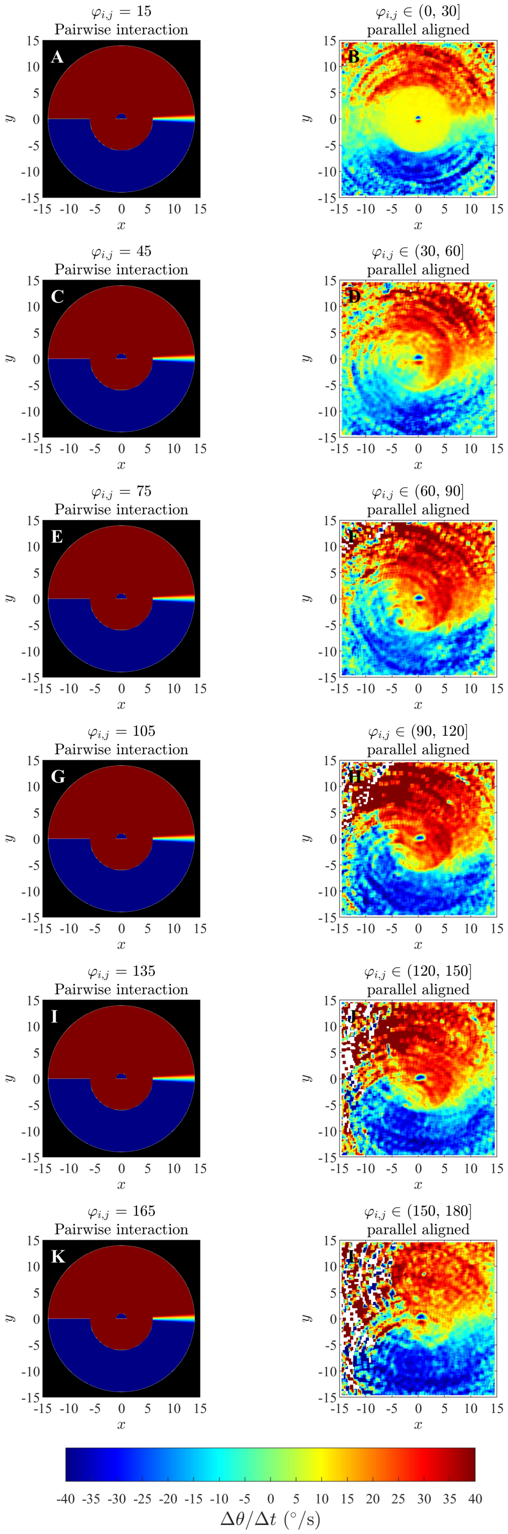

Fig S12. Left column: analytical pairwise changes in direction as prescribed by the zonal model with $r_r = 1; \Delta r_o = 5; \Delta r_a = 8; \varphi_{i,j} > 0$ and $\omega_{blind} = 0°$. Right column: results of force mapping.

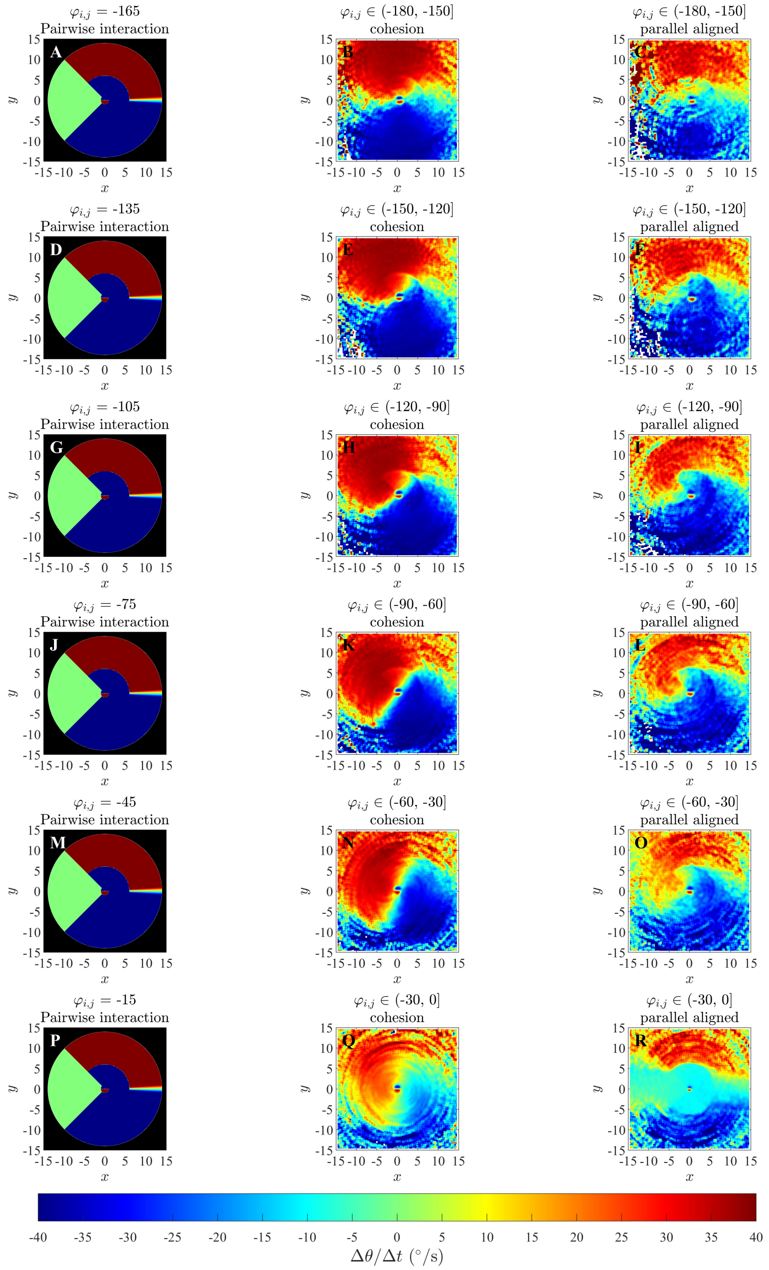

Fig S13. Left column: analytical pairwise changes in direction as prescribed by the zonal model with $r_r = 1; \Delta r_o = 5; \Delta r_a = 8; \varphi_{i,j} \leq 0$, $\omega_{blind} = 0°$ in ZOR and $\omega_{blind} = 90°$ in the ZOO and ZOA. Middle and right column: results of force mapping.

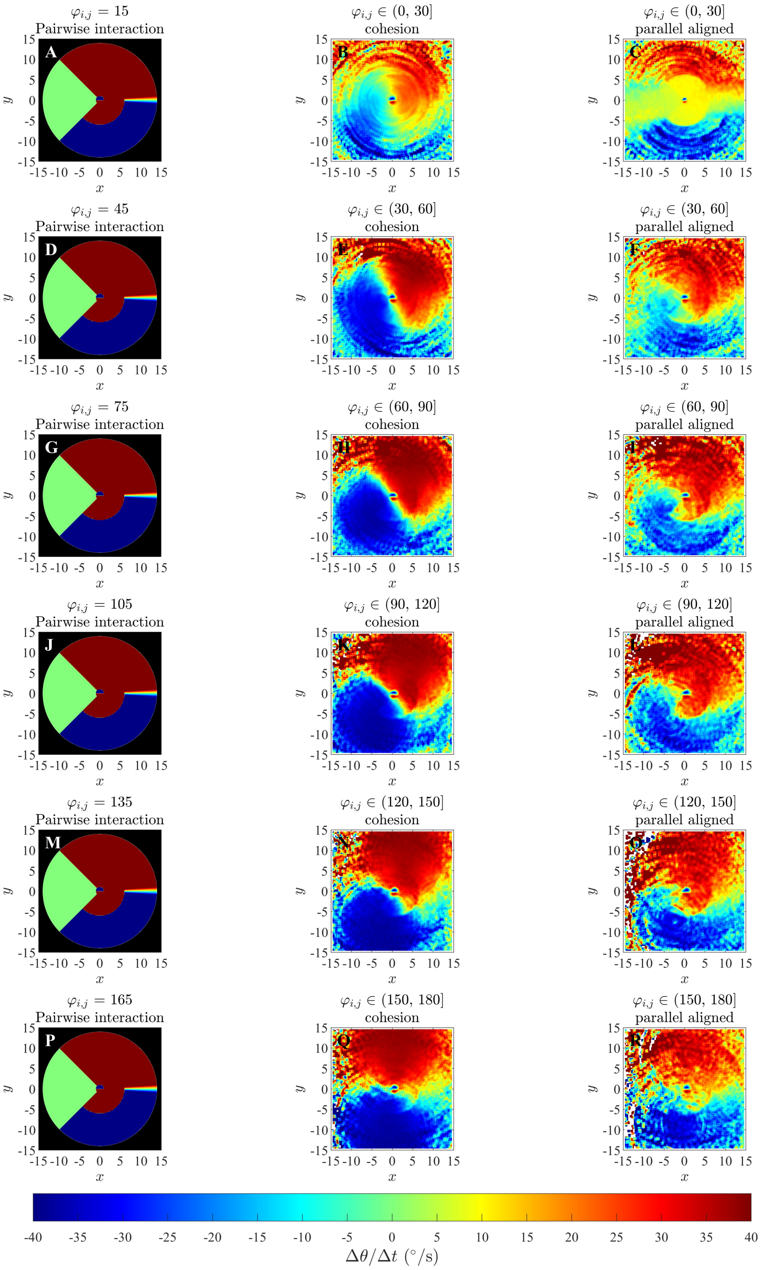

Fig S14. Left column: analytical pairwise changes in direction as prescribed by the zonal model with $r_r = 1; \Delta r_o = 5; \Delta r_a = 8; \varphi_{i,j} > 0$, $\omega_{blind} = 0°$ in ZOR and $\omega_{blind} = 90°$ in the ZOO and ZOA. Middle and right column: results of force mapping.

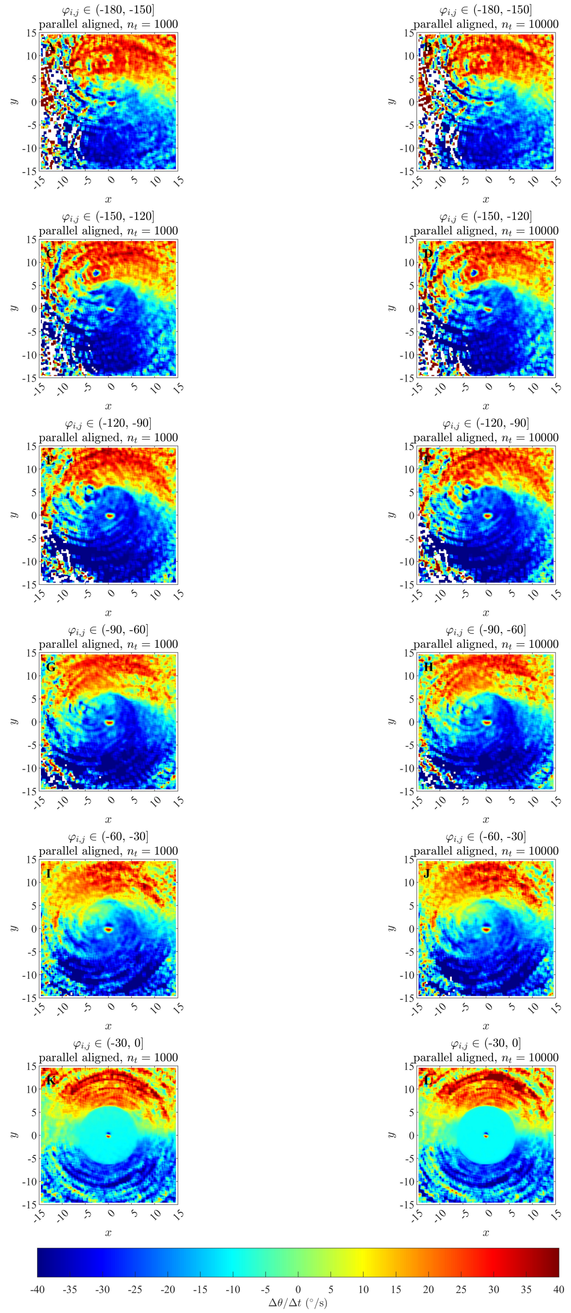

Fig S15. A comparison of analyses of simulations run for 1000 time steps (left column) and 10000 time steps (right column) when $r_r = 1; \Delta r_o = 5; \Delta r_a = 8; \varphi_{i,j} < 0$ and $\omega_{blind} = 0°$.

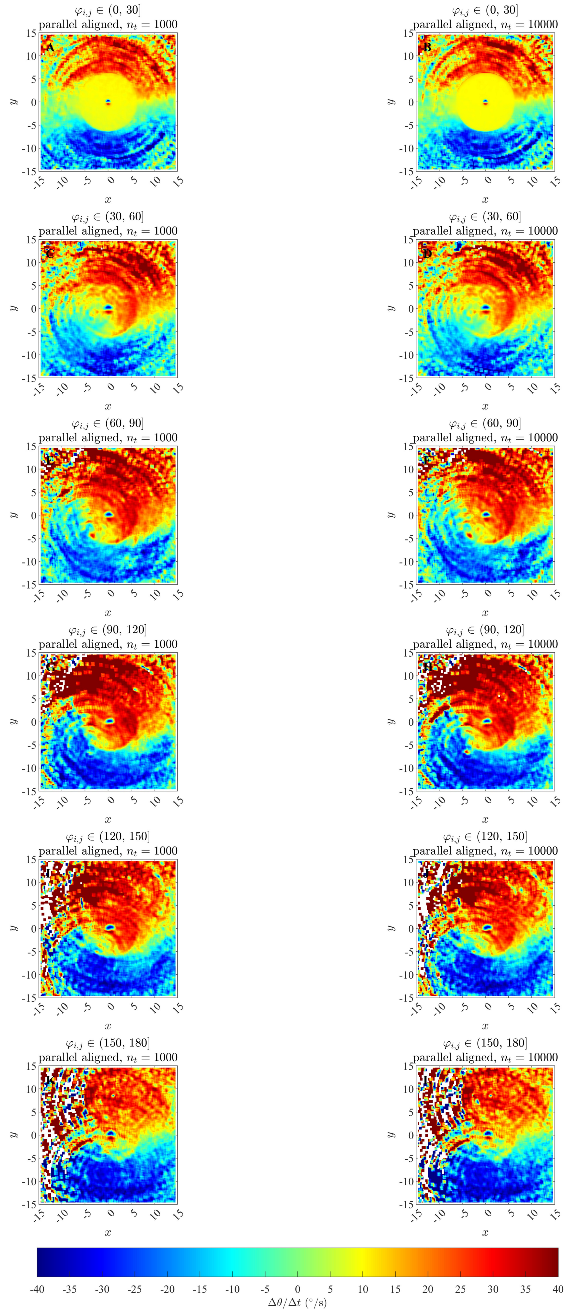

Fig S16. A comparison of analyses of simulations run for 1000 time steps (left column) and 10000 time steps (right column) when $r_r = 1; \Delta r_o = 5; \Delta r_a = 8; \varphi_{i,j} > 0$ and $\omega_{blind} = 0°$.

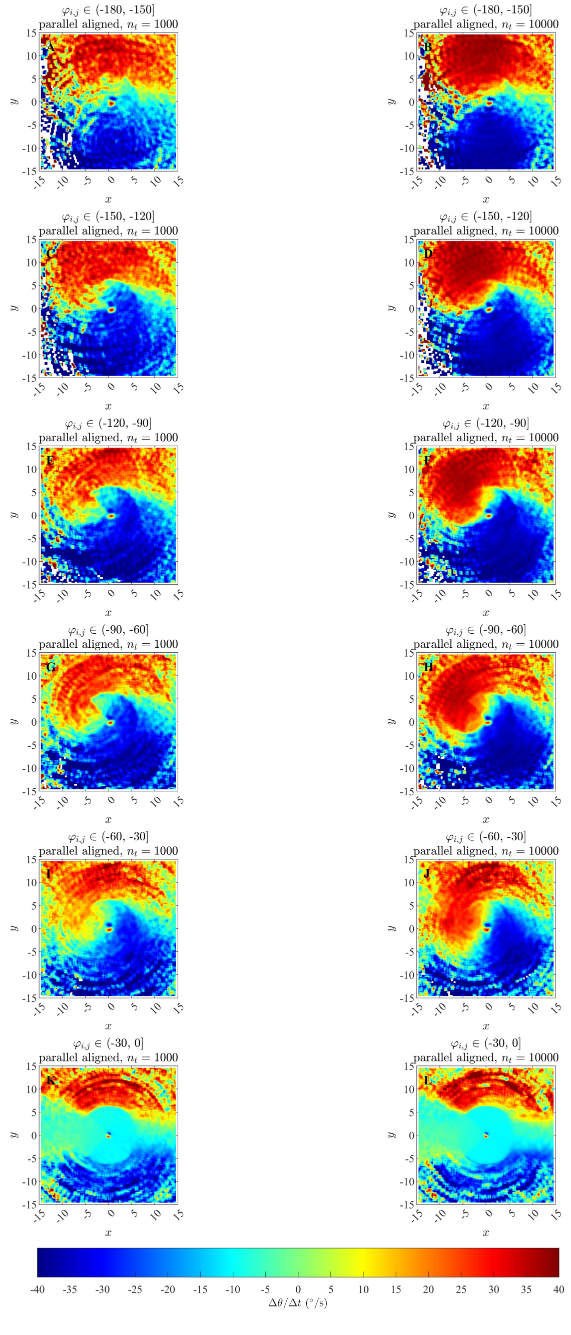

Fig S17. A comparison of analyses of simulations run for 1000 time steps (left column) and 10000 time steps (right column) when $r_r = 1; \Delta r_o = 5; \Delta r_a = 8; \varphi_{i,j} < 0$ and $\omega_{blind} = 90°$.

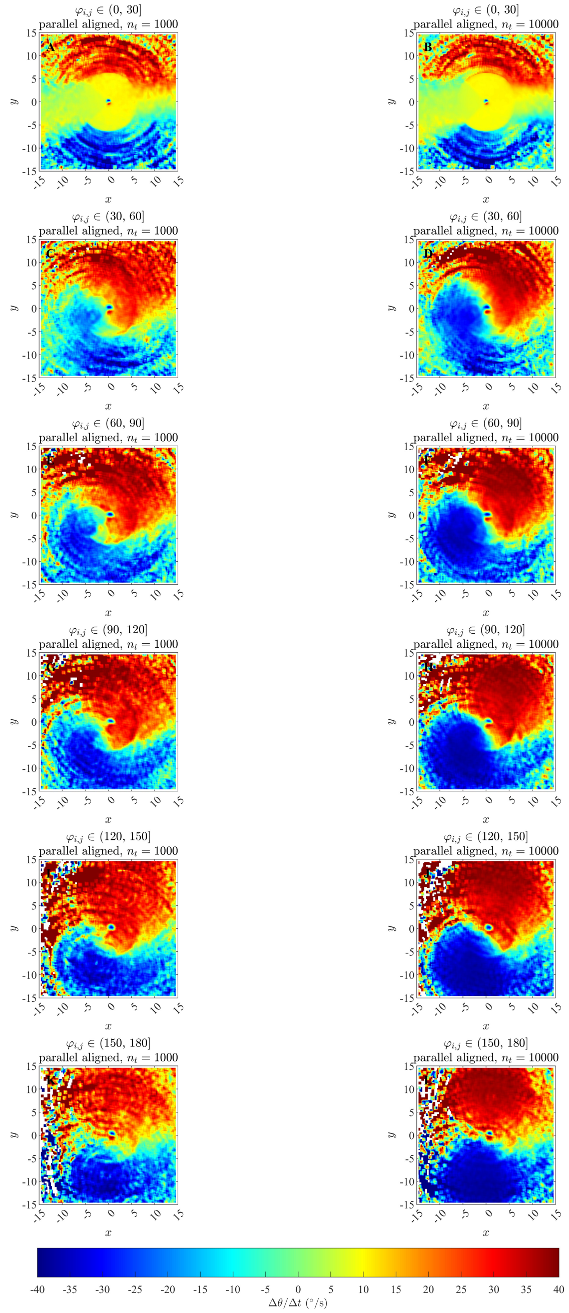

Fig S18. A comparison of analyses of simulations run for 1000 time steps (left column) and 10000 time steps (right column) when $r_r = 1; \Delta r_o = 5; \Delta r_a = 8; \varphi_{i,j} > 0$ and $\omega_{blind} = 90°$.

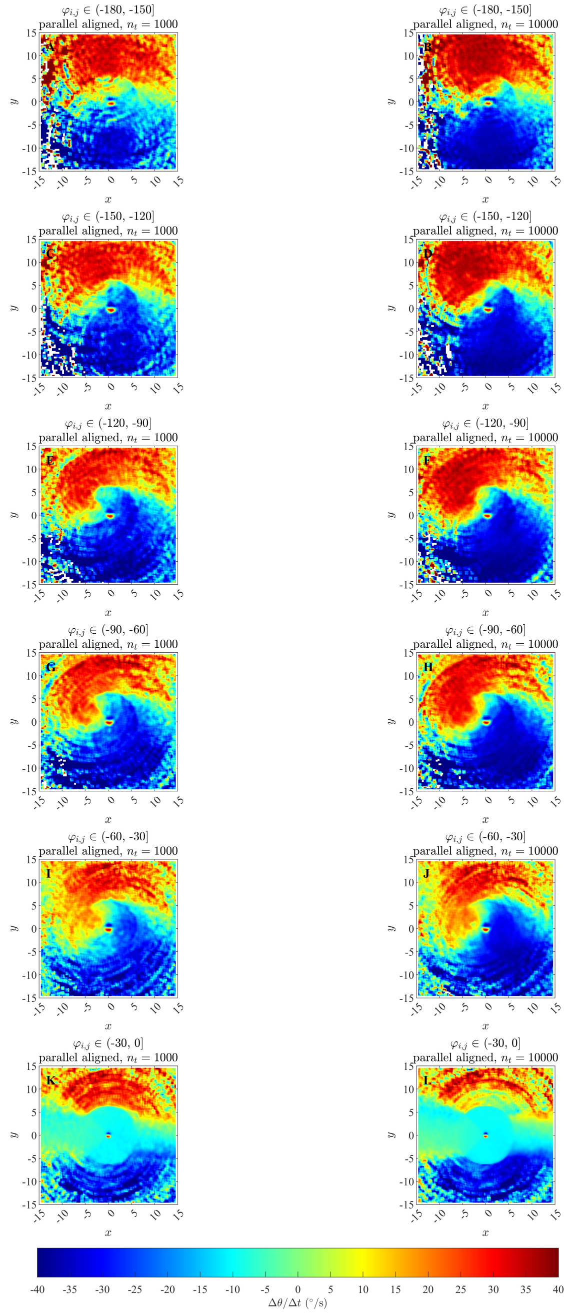

Fig S19. A comparison of analyses of simulations run for 1000 time steps (left column) and 10000 time steps (right column) when $r_r = 1$; $\Delta r_o = 5$; $\Delta r_a = 8$; $\varphi_{i,j} < 0$; $\omega_{blind} = 0°$ in ZOR and $\omega_{blind} = 90°$ in the ZOO and ZOA.

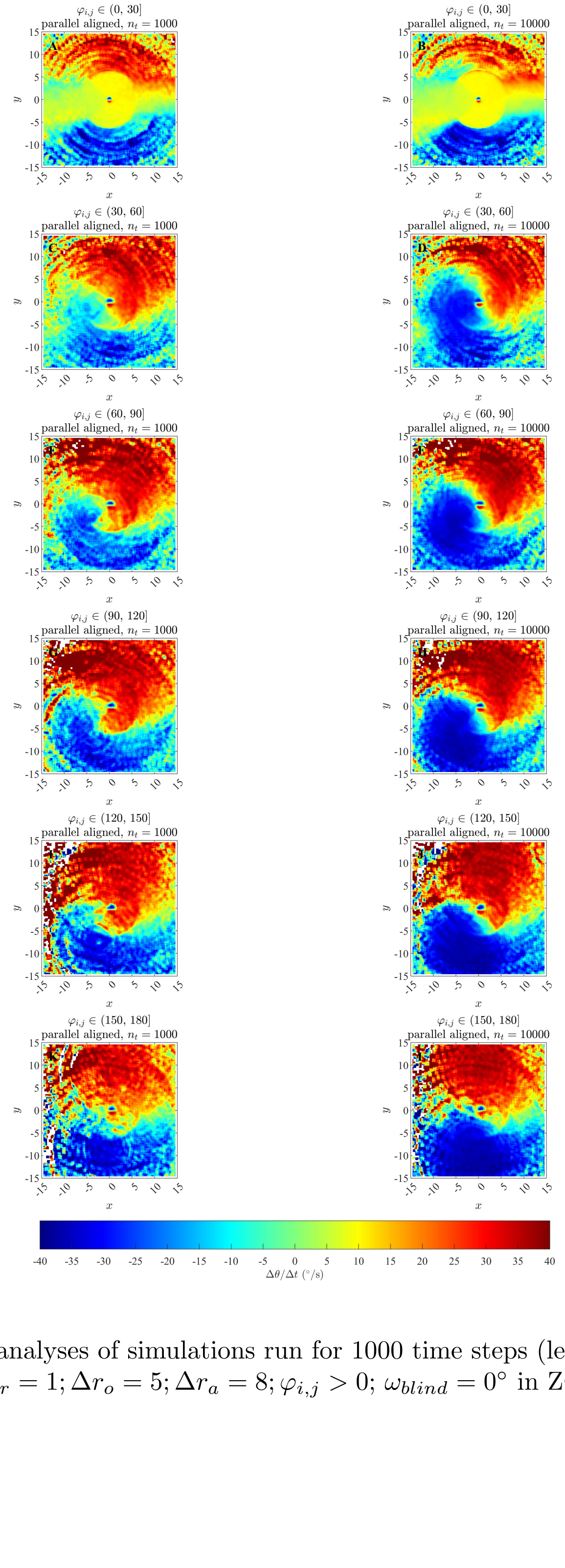

Fig S20. A comparison of analyses of simulations run for 1000 time steps (left column) and 10000 time steps (right column) when $r_r = 1; \Delta r_o = 5; \Delta r_a = 8; \varphi_{i,j} > 0; \omega_{blind} = 0°$ in ZOR and $\omega_{blind} = 90°$ in the ZOO and ZOA.

S3.3 Plots from analysis of ODE model

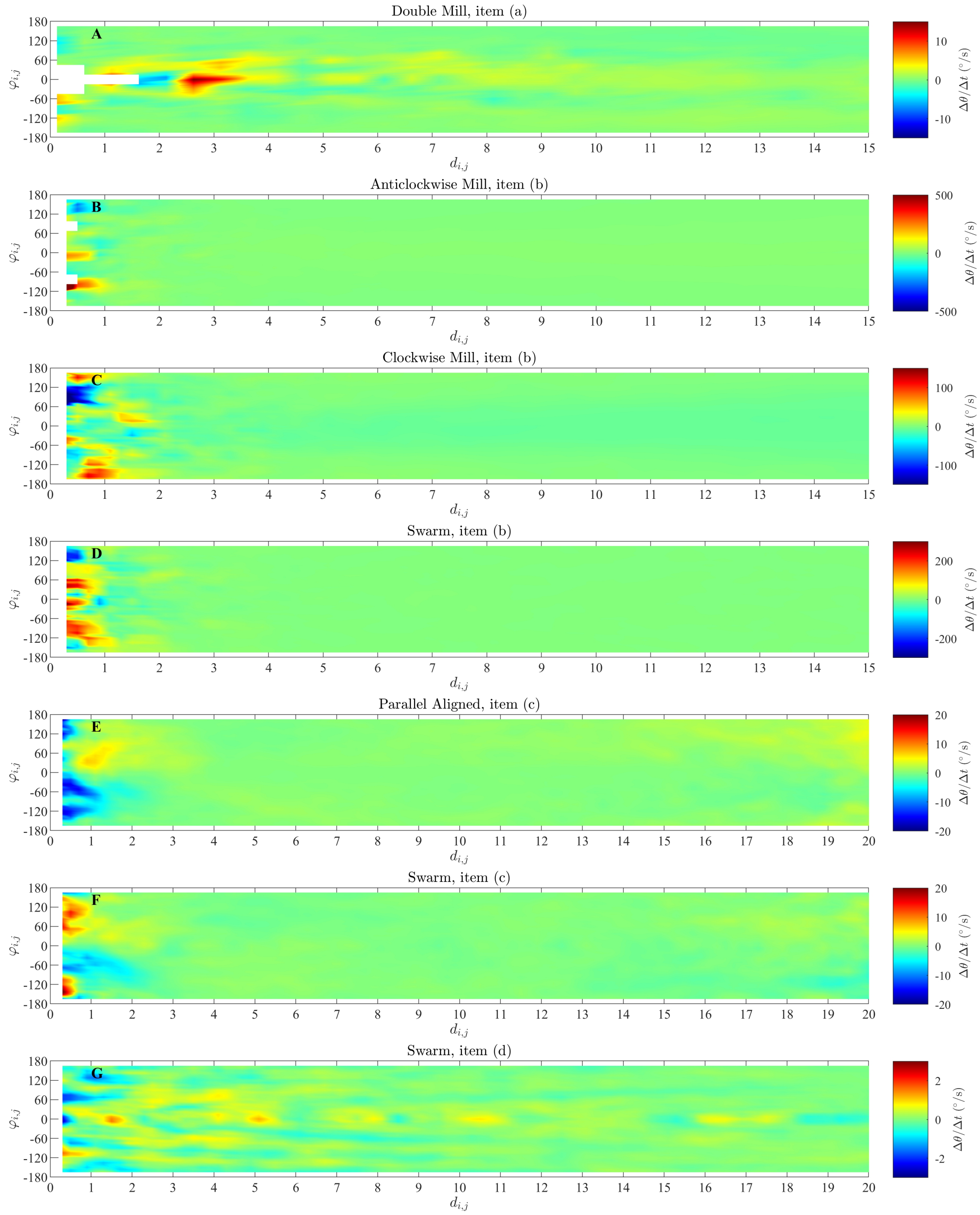

Fig S21. Panels A-G are respective plots for each item in Table S5. Mean changes in direction over time of individuals as a function of both distance to other group mates and the differences in directions of motion between a focal individual and group mate. Where angular differences, $\varphi_{i,j}$, and $\frac{\Delta\theta}{\Delta t}$ had like signs, focal individuals tended to align with their neighbours. Positive values of $\frac{\Delta\theta}{\Delta t}$ correspond to anti-clockwise turns, and negative values of $\frac{\Delta\theta}{\Delta t}$ correspond to clockwise turns.

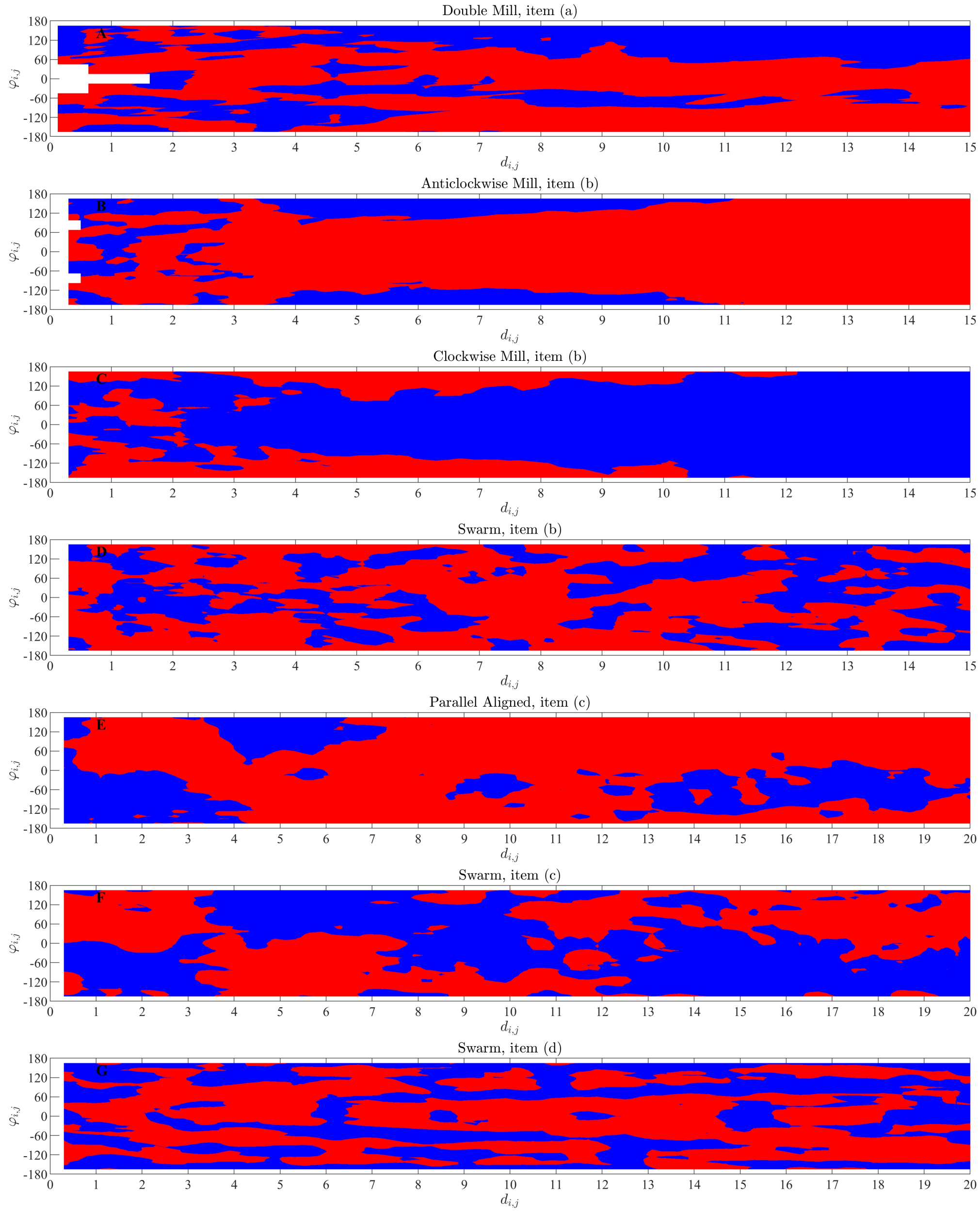

Fig S22. The sign of $\frac{\Delta\theta}{\Delta t}$ for the sequence of graphs illustrated in Figure S21. Red indicates an anticlockwise (positive) turn, whereas blue indicates a clockwise (negative) turn.

References

1. Couzin, I. D., Krause, J., James, R., Ruxton, G. D., and Franks, N. R. Collective memory and spatial sorting in animal groups. *Journal of Theoretical Biology 218*, 1 (2002), 1–11.

2. Davis, S., Lukeman, R., Schaerf, T. M., and Ward, A. J. W. Familiarity affects collective motion in shoals of guppies (*Poecilia reticulata*). *Royal Society Open Science 4*, 9 (2017), 170312.

3. D'Orsogna, M. R., Chuang, Y.-L., Bertozzi, A. L., and Chayes, L. S. Self-propelled particles with soft-core interactions: patterns, stability, and collapse. *Physical Review Letters 96*, 10 (2006), 104302.

4. Herbert-Read, J. E., Perna, A., Mann, R. P., Schaerf, T. M., Sumpter, D. J. T., and Ward, A. J. W. Inferring the rules of interaction of shoaling fish. *Proceedings of The National Academy of Sciences 108*, 46 (2011), 18726–18731.

5. Katz, Y., Tunstrøm, K., Ioannou, C. C., Huepe, C., and Couzin, I. D. Inferring the structure and dynamics of interactions in schooling fish. *Proceedings of The National Academy of Sciences 108*, 46 (2011), 18720–18725.

6. Mudaliar, R. K., and Schaerf, T. M. Examination of an averaging method for estimating repulsion and attraction interactions in moving groups. *PLoS ONE 15*, 12 (2020), e0243631.

7. Schaerf, T. M., Dillingham, P. W., and Ward, A. J. W. The effects of external cues on individual and collective behavior of shoaling fish. *Science Advances 3*, 6 (2017), e1603201.

8. Schaerf, T. M., Herbert-Read, J. E., and Ward, A. J. W. A statistical method for identifying different rules of interaction between individuals in moving animal groups. *Journal of the Royal Society Interface 18*, 176 (2021), 20200925.

9. Vicsek, T., Czirók, A., Ben-Jacob, E., Cohen, I., and Shochet, O. Novel type of phase transition in a system of self-driven particles. *Physical Review Letters 75*, 6 (1995), 1226.

10. Ward, A. J. W., Schaerf, T. M., Herbert-Read, J. E., Morrell, L., Sumpter, D. J. T., and Webster, M. M. Local interactions and global properties of wild, free-ranging stickleback shoals. *Royal Society Open Science 4*, 7 (2017), 170043.
