# Supplementary material for: An examination of force maps targeted at orientation interactions in moving groups
Source: PLoS One. 2023 Sep 7;18(9):e0286810. doi: 10.1371/journal.pone.0286810 (PMC10484433; doi:10.1371/journal.pone.0286810)
Supplement: Attachment — Submitted filename: review_Mudaliar_et_al.pdf [file pone.0286810.s003.pdf]

Dear editor,

please find below my detailed report concerning the article "An examination of force maps targeted at orientation interactions in moving groups" by R. K. Mudaliar and T. M. Schaerf, submitted for publication to PLOS One.

This work studies in great detail the method called *force mapping* (FM), which has been used in the past to infer the underlying interaction rules of individuals moving in groups, specially in shoals of fish.

The authors started by introducing previous works and related methods – like the *force matching* method –, and highlighted in detail the similarities and differences between those previous works and the FM method. After this introduction, the authors listed the limitations of the FM method and introduced the methodology they used to study it. This methodology consisted on using the FM method on trajectories of a group of continuously moving individuals that were obtained using three established models: M1) the Vicsek model [T. Vicsek et al., Physical Review Letters, 1995], M2) the three-zone model [I.D. Couzin et al., Journal of Theoretical Biology, 2002] and M3) an ODE model [M. R. D'Orsogna et al., Physical Review Letters, 2006]. Thus, the authors knew the model parameters and explored in how far the FM method could recover the most important features of the three models.

In Section 2, the authors proceeded to introduce the main relative variables of the study – like the change in the direction of motion $\Delta\theta/\Delta t$ – and explained how they aggregated the data to produce the heat maps of the FM method, that they presented in the Results section. These results are given and explained in great detail for each of the models, where they also studied how their results change by modifying the boundary conditions [bounded vs. unbounded conditions].

The authors concluded that the FM method is mainly capable to recover the aligned zones in case where these are symmetric to the origin [of the focal agent], but does not show such zones on data generated with models without explicit alignment interactions. They also mention that their analysis suggest that the FM method does not always reveal the social rules even when the dependent and independent variables of the method are chosen to exactly match the variables that define the behavioral rules in the models.

The paper is well written and the sections are well structured. The conclusions seem reasonable within the context of the study and I think that this could be potentially published in PLOS One once following questions and issues are addressed by the authors.

- **Q1)** I think the paper lacks of a clear, concise and succinct way of presenting the main results of the analysis presented in this work. What I mean is that a reader might not easily find the information on when the FM method provides a reliable way to infer properties of the social interactions of moving individuals. I know that this information is already in the paper, but it is rather scattered/distributed in the discussion, results and SI sections. Perhaps, the authors could provide a sort of list/table in the discussion section for this (not 100% sure this is the best option, perhaps the authors have a better idea). By doing so, I believe that the impact of this work might be increased.

- **Q2)** A second important point would be to relate the results provided here to real-life biological and/or synthetic systems. If a reader of this paper is working with, let's say, fish in a tank [bounded system], and another reader is working with moving groups of ungulates in the wild [unbounded system], what should they expect from

the FM method based on the results presented in this paper by the authors? This might be information that could be added/merged to the table of **Q1**, and I would also suggest to add this in the discussion section as well.

Q3) Why did the authors use two different group sizes for the results presented for the Vicsek model [they used $N = 10$ individuals] and the results for the zone model [they used $N = 25$ individuals]? Is there an important reason for doing so? If so, this reason should be discussed in the paper. If not, I would suggest to run new simulations and modify the plots to have only one system size across all the paper, which could be fixed, for example, to $N = 25$.

Q4) The size of the plots and labels of most of the figures of the paper are very small. I would suggest to increase the font size and the size of all the plots.

Q5) In Figure 6, the authors show how the area of the region high alignment of the Vicsek model changes with increasing interaction radius for different system sizes. They report a simple linear regression, but visually to me it looks that the dependence might be rather non-linear, perhaps with some exponent larger than 1. Did the authors implement other non-linear regression methods? If so, please comment on why they did not included them on the paper. If not, I would suggest the authors to do it and interpret the resulting exponent. Also, in the same figure, there seems to be a slight dependency on the system size. Could the authors please comment on this and what would this mean when studying real-life large systems? [see **Q1** and **Q2**].

Q6) Finally, do the authors have some concrete suggestions to improve the FM method? For example, proposing a new way to aggregate data to produce more informative heat maps? For this, no exhaustive study is needed but rather the authors could use the knowledge they got from using the FM method to propose improvements as an outlook of the whole paper.
